# Multi-Country Study of Stable Isotopes and Mineral Elements in European Pork

**DOI:** 10.3390/foods15081317

**Published:** 2026-04-10

**Authors:** Anna Pinna, Rosaria Fragni, Roberta Virgili, Cecilia Loffi, Isabel Revilla, Ana M. Vivar-Quintana, Ewa Sell-Kubiak, Agnieszka Ludwiczak, Anita Zaworska-Zakrzewska, Marchen Sonja Hviid, Carolina Reyes-Palomo, Santos Sanz-Fernández, Andrea Bertolini, Anna Garavaldi, Paolo Ferrari

**Affiliations:** 1 SSICA, Experimental Station for the Food Preserving Industry—Research Foundation, 43121 Parma, Italy; rosaria.fragni@ssica.it (R.F.); roberta.virgili@ssica.it (R.V.); cecilia.loffi@ssica.it (C.L.); 2 Area of Food Technology, Polytechnical High School of Zamora, University of Salamanca, 49022 Zamora, Spain; irevilla@usal.es (I.R.); avivar@usal.es (A.M.V.-Q.); 3 Department of Genetics and Animal Breeding, Poznan University of Life Sciences, Wołyńska 33, 60-634 Poznań, Poland; ewa.sell-kubiak@puls.edu.pl; 4 Department of Animal Breeding and Product Quality Assessment, Poznan University of Life Sciences, Szydłowska 50, 60-627 Poznań, Poland; agnieszka.ludwiczak@puls.edu.pl; 5 Department of Animal Nutrition, Poznan University of Life Sciences, Wołyńska 33, 60-637 Poznań, Poland; anita.zaworska-zakrzewska@up.poznan.pl; 6 Danish Meat Research Institute, Danish Technological Institute, DK-2630 Taastrup, Denmark; mahd@teknologisk.dk; 7 Department of Animal Production, International Agrifood Campus of Excellence (ceiA3), University of Córdoba, Campus de Rabanales, 14071 Córdoba, Spain; v22repac@uco.es (C.R.-P.); v22safes@uco.es (S.S.-F.); 8 CRPA, Research Centre for Animal Production s.c.p.a., 42121 Reggio Emilia, Italy; a.bertolini@crpa.it (A.B.); a.garavaldi@crpa.it (A.G.); p.ferrari@crpa.it (P.F.)

**Keywords:** European pork, stable isotopes, mineral elements, traceability, geographical origin, authenticity

## Abstract

European pork production pursues traceability and authenticity to ensure animal welfare, food safety, and support products with geographical indications. This study reports a European survey integrating stable isotope ratios (δ^13^C, δ^15^N, δ^34^S, δ^18^O, δ^2^H) and multi-element profiling using IRMS and ICP-MS, on 612 samples collected across Denmark, Poland, Italy, and Spain, with diverse production systems, breeds, feeding, and slaughter ages. Geographical and climatic gradients influenced δ^2^H and δ^18^O, which ranged from −111‰ to −89‰ in samples from Denmark and Spain and from 13.3‰ to 16.0‰ in samples from Italy and Spain, respectively. In selected farms, δ^13^C ranged from −22.7‰ to −17.0‰ depending on diet composition based on C_3_ and C_4_ plants. The wide variability in pig management practices suggested that δ^15^N (2.50 ÷ 4.96‰) increased with slaughter age and was positively correlated with Fe (3.38 ÷ 8.39 mg/kg) and Zn (9.39 ÷ 23.6 mg/kg). Most mineral components were mainly driven by feed formulation and supplementation. Principal component analysis (PCA) showed that samples were grouped based on their origin and husbandry system, confirming the key role of isotopic and elemental markers for the development of a database supporting the pork supply chains across Europe.

## 1. Introduction

Pork represents one of the most important animal-derived foods worldwide and plays a central role in human nutrition, providing high-quality proteins, essential fatty acids, and key micronutrients [1]. Its consumption in the EU is around 40 kg per capita per year [2], and the EU is the world’s second-largest pork producer and the largest exporter of pork and pork products [3,4]. As a result, authenticity and traceability have become key issues in the European meat sector, particularly for high-value products protected under Protected Designation of Origin (PDO) and Protected Geographical Indication (PGI), or certified as organic farming or free-range finishing products. Therefore, clear identification of origin and production practices is essential to meet consumer expectations, safeguard product reputation, prevent fraud, and ensure fair competition [5].

Pig production in Europe is highly heterogeneous, reflecting the diversity of regional traditions, environmental conditions, feeding systems, and breeds [5]. In Northern Europe, pigs are predominantly reared indoors in intensive systems and fed cereal-based diets based on C_3_ crops (wheat, barley, and rye). In contrast, production systems in Southern Europe may also rely on maize, a C_4_ crop, as a key dietary energy source, and traditional extensive production systems are common in these regions. In fact, in the Iberian Peninsula, traditional free-range systems are practiced in the Dehesa, where pigs graze outdoors and are fattened only on acorns and natural pasture during the final fattening phase (called Montanera in Spanish, meaning pannage), which takes place in autumn-winter after the ripening of *Quercus acorns* [6,7,8]. These diverse production systems correspond to different breed strategies. Intensive farming typically uses fast-growing commercial hybrids. In contrast, extensive and semi-extensive systems rely on local autochthonous breeds, which have longer rearing periods and are often linked to specific products with distinctive meat quality traits [9,10].

To support authenticity and traceability in meat products, advanced analytical methods have been increasingly adopted to characterize intrinsic compositional features that reflect geographical origin, diet, and production systems. Stable isotope ratio analysis (SIRA) and multi-element analysis, which, respectively, assess the isotopic ratios of light elements (C, N, H, O, and S) and the mineral element profile, have proved to be among the most effective techniques to determine the geographical origin of food products [11] and meat [12,13,14,15]. The isotopic composition in animal tissues is influenced by diet, environmental conditions, and metabolic processes. For example, carbon isotope ratios (δ^13^C) reflect the relative contribution of C_3_ versus C_4_ plants in the diet, whereas hydrogen (δ^2^H) and oxygen (δ^18^O) isotope signatures are primarily influenced by geographical and climatic differences in feed and water sources. Nitrogen (δ^15^N) values have been related to feed composition and agricultural practices [16,17]; however, evidence suggests that diet alone is not sufficient to explain δ^15^N variability, as animals fed similar diets in different geographical contexts may exhibit significantly different δ^15^N signatures in muscle tissue [18]. Sulfur isotope ratios (δ^34^S) in animal tissues originate from plant sulfur incorporated through the diet, which in turn reflects local soil and environmental factors, including geology, sulfide availability, microbial activity, fertilization, and atmospheric deposition (e.g., sea spray effect) [13]. Beyond isotopes, the elemental profile in muscle tissue reflects the geological and environmental context of production and differences in diet and farming practices. Mineral element content depends on feed, drinking water, pollution, and soil composition, all of which are linked to geographical origin [19]. Furthermore, husbandry practices including the strategic use of mineral supplementation—routinely incorporated into swine diets to support growth, skeletal development, and metabolic functions—can significantly influence the overall mineral profile and bioavailability within the animal [20]. Multivariate statistical methods, such as principal component or discriminant analysis, applied to SIRA and multi-element analysis, proved to be adequate tools for assessing meat provenance and authenticity [5,11,21].

To the best of our knowledge, only a limited number of studies have applied stable isotope ratio and/or mineral profile analysis to pork to date. Available research has mainly compared pork from different regions, including China [17,22], Romania and abroad [23,24], Korea and abroad [25,26,27,28]. Other studies investigated the effect of husbandry practices on isotopic and elemental profiles: organic versus conventional production [17], traditional Mediterranean pig production systems including Iberian pigs [29,30], a local Italian breed [31], and different feeding regimes within PDO supply chains in northern Italy [32]. However, no study to date has combined isotopic and elemental markers at a European scale, simultaneously covering multiple countries, diverse production systems, and fully documented husbandry conditions.

Our study addresses this gap and investigates pork origin, identifying stable isotope ratios and multi-element profiles, through mass spectrometry techniques (IRMS and ICP-MS), drawing on one of the largest European datasets to date. The study was conducted within the framework of the “mEATquality” EU project to provide reliable tools to explore the authenticity and traceability of pork meat obtained under both intensive and extensive production systems in Europe. The samples analyzed in the present study originated from Denmark, Poland, Italy, and Spain. In contrast to earlier studies based on specific regional sampling or single-marker approaches [12,13,14], the dataset comprises more than 600 samples with documented husbandry conditions, allowing an assessment of the combined effects of geography, diet (C_3_ and C_4_ plant composition), slaughter age, and production system—factors often examined in isolation [17,29,31]. The objectives of the study are as follows:

1. To improve understanding of the relationships between some of the above-mentioned husbandry practices, isotope ratios, and mineral profile of pork;
2. To evaluate the suitability of SIRA and multi-element analysis for identifying pork based on geographical origin and animal management, thus promoting the authenticity and traceability of European pork products;
3. To provide a basis for the development of a European reference database of isotopic and elemental data on pork, enabling the authentication, traceability, and protection of European pork products, including the eligibility for PDO and PGI designations.

## 2. Materials and Methods

### 2.1. Sample Origin

A total of 612 pork samples were collected from pigs reared on 12 farms located in Denmark, Poland, Italy, and Spain, adopting different production systems, breeds, and feeding regimes. Information about pork samples is summarized in Table 1. Alphanumeric codes were used to identify the farms, in which the first letter indicates the country of provenance and the production system (lowercase for intensive, uppercase for extensive), and the following number identifies the specific farm.

Denmark. Samples (n = 80) were collected from Duroc × (Landrace × Yorkshire) [DU × (LA × Y)] pigs, reared in one farm (d1), located in the central region operating under an intensive system, and supplying a cereal-based (C_3_) feeding regimen, based on wheat, barley, and soybean meal.

Poland. Samples (n = 118) originated from three farms (p1, p2, and p3) in the Greater Poland Voivodeship (western-central Poland), which adopted intensive husbandry systems. One trial in p1 included three pig lines (each group n = 10), namely Polish Large White (LW), Polish Large White × Pulawska (LW × PLW), and Pulawska (PLW), reared under a C_3_ feeding regimen based on barley, rye, and wheat bran. A second trial was carried out in the same farm with PIC^®^ pigs [33] (n = 30). Farm p2 supplied (Polish Large White × Polish Landrace) × (Duroc × Pietrain) pigs [(LW × LA) × (DU × P)] fed with a C_3_ feeding regimen composed of barley, wheat, and triticale (n = 30). Farm p3 provided Pulawska (PLW) pigs fed with C_3_ plants: peas, oat protein, and buckwheat (n = 30).

Italy. Samples (n = 210) were collected from pigs reared on four farms (I1, I2, i3 and i4), located in the northern and central regions, applying semi-extensive and intensive husbandry systems. Farm I1, placed in Emilia-Romagna, reared Large White × Duroc (LW × DU) pigs (n = 10) and the local breed Mora Romagnola (MR) (n = 10), fed with wheat, maize, and soy (C_3_ and C_4_ diet), under a semi-extensive system. Farm I2, located in Tuscany, reared a PDO Italian local breed Cinta Senese (CS); additionally, the crossbreeds Duroc × Cinta Senese (DU × CS) and Large White × Cinta Senese (LW × CS) were included in the trial. All animals were fed a C_3_ and C_4_-based diet composed of maize, barley, and sorghum, under a semi-extensive outdoor system (n = 30). Farm i3, located in Emilia-Romagna, reared Italian Duroc × Large White (DU × LW) pigs, fed a C_3_- and C_4_-based diet under an intensive system (n = 60). Farm i4 located in Piedmont, reared Topigs^®^ [34], TN70 × Fomeva K-line pigs [Topigs Large White × (Large White × Landrace)], fed a C_3_ organic diet produced mainly on the farm, composed of barley and wheat, in an intensive system; a total of 100 pigs were obtained for the trial in four replicas. The raw matter obtained from pigs reared in these farms can be used for the production of Italian PDO products, provided that the production regulations are respected.

Spain. A total of 204 samples were collected from four farms (S1, S2, s3 and s4), which employed both extensive and intensive systems. Three farms were in Andalucia (S1, S2, and S3) and the other in Murcia (s4). In the S1 farm, the Spanish autochthonous Iberian (IB) and Iberian × Duroc (IB × DU) pigs were reared according to the extensive Montanera system [35], in which the free-range fattening phase takes place on Dehesa pasture and the animals feed exclusively on *Quercus* acorns and grasses for more than two months [6]; IB (n = 17) and (IB × DU) (n = 17) pigs were included in the trial. On the same farm, IB × DU pigs were reared under an intensive system (s1), fed a C_3_- and C_4_-based diet (n = 34). In farm S2, IB and IB × DU pigs (n = 34) were reared under the extensive Cebo de Campo system [35], an intermediate diet regimen in which animals graze on acorns and grasses but receive supplemental C_3_-based feed. In farm s3, IB pigs were reared under an intensive system, and fed a C_3_-based diet (n = 34). In farm s4, Landrace × Large White (LA × LW) pigs were reared under an intensive system, and fed with corn, barley, and soy flour (n = 68). Products obtained by Iberian pigs may be protected under PDO labels. In S1 IB, pigs were labeled as “100% Ibérico bellota” IBxDU as “Ibérico bellota”. In S2, pigs were classified in the “Cebo de Campo Ibérico” category, whereas pigs from farms s1 and s3 were classified in the “Cebo Ibérico” category.

#### Sampling

Pork *longissimus thoracis et lumborum* (LTL) samples were collected directly at the abattoir from each carcass and delivered to the laboratories in Italy (SSICA), Spain (USAL), Denmark (DTI), and Poland (PULS), within 48 h post-mortem. At the laboratory, a 5 cm-thick slice was taken from the T7–T9 section of the LTL muscle. A portion of the slice was immediately analyzed for moisture and fat content according to AOAC Official Method [36,37], whereas the remaining sample was vacuum-packed and stored at −20 °C, and then freeze-dried to obtain the dry matter fraction (DM). In a portion of the DM, the fat was removed via Soxhlet extraction to obtain the defatted freeze-dried matter (DFDM) [38]. To ensure inter-laboratory comparability, the protocols were defined and shared among laboratories prior to sample analysis. Quality control was further ensured by laboratory accreditation under ISO/IEC 17025 [39] and participation in annual proficiency testing schemes (SSICA and DTI), or analysis of NIST standard reference material 1546a (SSICA, USAL, and PULS).

Collected DM and DFDM were shipped to SSICA for the analysis of mineral elements and δ^13^C, δ^15^N, and δ^34^S analysis, whereas another fraction of DFDM was shipped to AgroIsolab GmbH (Germany) for δ^2^H and δ^18^O analysis.

### 2.2. Stable Isotope Ratio Analysis (δ^13^C, δ^15^N, δ^34^S, δ^18^O, and δ^2^H)

The analysis of δ^13^C, δ^15^N, and δ^34^S was performed according to Perini et al. [38] using an elemental analyser (Vario PYRO Cube, Elementar, Langenselbold, Hesse, Germany) coupled to an Isotope Ratio Mass Spectrometer (IRMS, Precision, Elementar, Langenselbold, Hesse, Germany). DFDM LTL samples (1.5 mg) were weighed into tin capsules and introduced via an auto-sampler. Combustion occurred at 1150 °C in the presence of oxygen, converting C, N, and S into CO_2_, N_x_, and SO_2_. NO_x_ was reduced to N_2_ in a copper reduction column at 850 °C. Gases were sequentially trapped and released (CO_2_ at 110 °C, SO_2_ at 220 °C), and analyzed by IRMS. Samples were analyzed in duplicate, and values accepted when precision was <0.02 for δ^13^C, <0.03 for δ^15^N, and <0.05 for δ^34^S. The calibration of isotopic values was performed using the following international standard: Tibetan Human Hair (USGS-42) (δ^15^N = +8.05‰, δ^13^C = −21. 09, δ^34^S = +7.84‰); Caffeine (USGS-61) (δ^15^N = −2.87‰, δ^13^C = −35.05); L-Valine (USGS-74) (δ^15^N = 30.19, δ^13^C = −9.30) and Barium sulfate IAEA-SO-5 (δ^34^S = +0.5 ± 0.2‰VCDT). δ^18^O and δ^2^H measurements were performed at AgroIsolab GmbH using two Isotope Ratio Mass Spectrometers (IRMS) in a master/slave configuration (Nu Instruments, Wrexham, North Wales, UK) with each IRMS measuring one isotope ratio (δ^18^O or δ^2^H). Samples were weighed in tin cartridges (4 × 6 mm) and analyzed in pyrolysis mode at 1550 °C using a patented silicon carbide tube (Agroisolab GmbH, Jülich, Germany) filled with glassy carbon chips and coal powder connected to a Zero-blank autosampler (ZB-REV2.0, AIL-Tech/Hekatech, Jülich, Germany) equipped with a heated drying chamber maintained at 60 °C under continuous helium flow (20 mL/min) to prevent Maillard reactions and residual water contamination. To address the known issue of exchangeable hydrogen and oxygen in organic matrices, all samples were equilibrated for 24 h in a humidity chamber at a defined relative humidity (10%) using a laboratory water of known isotopic composition (δ^18^O = −7.8‰), followed by storage in a vacuum chamber (<100 mbar) for at least 24 h prior to analysis. This equilibration procedure ensures that the exchangeable fraction of hydrogen and oxygen reaches isotopic equilibrium with the laboratory water before measurement, allowing its contribution to be controlled and accounted for in the final isotopic values. The measurement run was continuously monitored using the laboratory standard dihydroxyanthraquinone, calibrated against international reference materials, in a sequence of six standards at different weighing amounts to control and normalize the intensity non-linearity effect. Two secondary standards (Benzoin and Leucine) with a δ^2^H difference of approximately 300‰ were used to verify the scaling of the δ^2^H axis. The Leucine standard was additionally used to assess any influence of nitrogen on the δ^18^O signal. A routine sample was included in each run to verify the consistency of results over time. Calibration was performed against the following international standards: δ^18^O: IAEA-601, IAEA-602, and USGS-54; δ^2^H: IAEA-CH7, USGS-77, and USGS62. All laboratory standards are further verified through Agroisolab’s internal round-robin test scheme (KPT), with currently over 30 participating laboratories.

The mode Combined Uncertainty for δ^18^O and δ^2^H was 0.2‰ and 1.3‰, respectively.

Isotope ratios were converted into delta notation (δ‰) by using Equation (1):

(1) $$\delta_{ref}‰=\left(\frac{R_{sample}-R_{reference}}{R_{reference}}\right)\times 1000$$

where *R_sample_* and *R_reference_* are the absolute isotope ratios of the sample and reference material, respectively, and δ*_ref_*‰ is the deviation of the isotopic ratio of the sample relative to the reference material, expressed in delta units (‰, per mil). δ^13^C, δ^15^N, δ^34^S, δ^18^O, and δ^2^H values are reported in conventional δ notation relative to Vienna Pee Dee Belemnite (VPDB) for carbon, atmospheric nitrogen (AIR) for nitrogen, Vienna Canyon Diablo Troilite (VCDT) for sulfur, and Vienna Standard Mean Ocean Water (VSMOW) for oxygen and hydrogen.

### 2.3. Multi-Element Analysis

#### 2.3.1. Chemicals and Reagents

For sample digestion, ultra-pure grade concentrated nitric acid (69%, *m*/*v*; Ultrex™ II, J.T. Baker™, Avantor, Radnor, PA, USA) and nitrogen (5.0 grade) of 99.9990% purity (SIAD S.p.A., Bergamo, Italy) were used. All solutions introduced into the ICP-MS were prepared in graduated polypropylene (PP) tubes (DigiTUBEs, SCP Science, Champlain, NY, USA) or polymethylpentene (PMP) volumetric flasks (Brand GmbH, Wertheim am Main, Wertheim, Germany), using high-purity deionized water (0.05 μS/cm; Purelab Ultra, ELGA) for all dilutions. Membrane filters (0.45 µm; Millex^®^-HA, Millipore, Merck, Darmstadt, Germany) were employed for filtering mineralized samples. Customized multi-element stock solution containing Ca 1000 mg/L; K 1000 mg/L; Mg 1000 mg/L; Na 1000 mg/L; P 1000 mg/L; Ag 1 mg/L; As 1 mg/L; Ba 1 mg/L; Cd 1 mg/L; Co 1 mg/L; Cr 1 mg/L; Cs 1 mg/L; Cu 10 mg/L; Fe 10 mg/L; Ga 1 mg/L; Li 1 mg/L; Mn 1 mg/L; Mo 1 mg/L; Ni 1 mg/L; Pb 1 mg/L; Rb 1 mg/L; Se 1 mg/L; Sr 1 mg/L; Tl 1 mg/L; V 1 mg/L; Zn 50 mg/L; U 1 mg/L in 10% HNO_3_ (CPA chem, Stara Zagora, Bulgaria) was opportunely diluted to prepare the calibration curves in 1% HNO_3_. Be, Bi, Rh, and Sc mono-element stock solutions (1000 mg/L; CPA chem, Stara Zagora, Bulgaria) were used as internal standards to correct instrumental drift and matrix effects; Be, Bi, Rh were diluted 1000-fold prior to being added both to calibration standards and mineralized samples. Instrument tuning and masses calibration were carried out with solutions containing 25 elements (Al 10 μg/L; Ag 6 μg/L; Ba 4 μg/L; Be 35 μg/L; Bi 3 μg/L; Ce 3 μg/L; Co 8 μg/L; Cs 3 μg/L; Cu 15 μg/L; Ga 10 μg/L; Ho 3 μg/L; In 3 μg/L; Li 8 μg/L; Mg 10 μg/L; Mn 6 μg/; Ni 15 μg/L; Rh 3 μg/L; Sc 8 μg/L; Sr 5 μg/L; Ta 3 μg/L; Tb 3 μg/L; Tl 4 μg/L; U 3 μg/L; Y 3 μg/L; Zn 20 μg/L) in 2% HNO_3_ (CPA chem, Stara Zagora, Bulgaria), and 7 elements (1 μg/L each of Ba; Bi; Ce; Co; In; Li; U) in 2.5% HNO_3_ and 0.5% HCl (CPA chem, Stara Zagora, Bulgaria), respectively. During ICP-MS analyses, Argon (5.5 grade) of 99.9995% purity and He (6.0 grade) of 99.9999% purity (SIAD S.p.A., Bergamo, Italy) were employed.

#### 2.3.2. Samples Preparation and Instrumentation

About 0.3 g of freeze-dried pork samples were directly weighed into quartz tubes and mineralized in triplicate with 2 mL of 69% (*v*/*v*) HNO_3_, using a high-pressure microwave-assisted digestion system (Ultrawave, Milestone s.r.l., Sorisole, Bergamo, Italy) pressurized with nitrogen to 50 bar. The mineralization program consisted of heating to 250 °C at 1500 W and 150 bar for 20 min, followed by a 15 min holding step at the same temperature and pressure, cooling to 60 °C, and controlled pressure release at 3 bar min^−1^. In each run, two procedural blanks were processed under identical conditions. The digested solutions were transferred to PP tubes, spiked with internal standards (Bi, Be, Rh at 1 mg/L and Sc at 1 mg/L final concentration), diluted to 50 mL with deionized water, and filtered through 0.45 µm membrane filters. The quantitative analysis of 27 mineral elements (Li, Na, Mg, P, K, Ca, V, Cr, Mn, Fe, Co, Ni, Cu, Zn, Ga, As, Se, Rb, Sr, Mo, Ag, Cd, Cs, Ba, Tl, Pb, and U) was performed by single-quadrupole inductively coupled plasma mass spectrometry (Q-ICP-MS; iCAP™ RQ, Thermo Fisher Scientific, Bremen, Germany), equipped with a QCell™ flatapole collision/reaction cell operated in helium collision mode for polyatomic interference removal. Sample introduction was performed via a peristaltic pump connected to a cyclonic spray chamber, a MicroMist nebulizer, and a 180-position autosampler (CETAC, mod. ASX-560, Teledyne Technologies, Omaha, NE, USA). Instrument operating parameters and measurement conditions are reported in Table 2.

Instrument performance was verified daily by monitoring sensitivity, signal stability, mass calibration, and plasma conditions (CeO^+^/Ce^+^ and Ba^2+^/Ba^+^ ratios) according to the manufacturer’s specifications. Element quantification was based on an external eight-point calibration curve covering concentration ranges of 0.5–20 mg/L for macroelements and 0.05–1000 mg/L for microelements. Instrument control and data processing were performed using the Thermo Fisher Scientific ICP-MS software (QTegra™, v. 2.10.4345.136). All samples were analyzed in batches, with the corresponding digestion blanks included in each batch. Calibration standards of known concentration were analyzed between samples to monitor instrumental stability and signal drift. Table 3 summarizes the isotopes monitored for each element, along with the method’s analytical performance parameters, including instrumental limits of detection (LODs) and quantification (LOQs), repeatability, and accuracy.

The multi-element method was validated following Fragni et al. (2018) [40]. Briefly, LODs and LOQs were calculated from ten consecutive measurements of the calibration blank. Linearity was confirmed across the full calibration range for all analytes. Method precision was assessed as short-term repeatability, expressed as relative standard deviation (RSD%), based on six consecutive measurements of a multi-element standard solution (1.0 mg/L for macroelements; 1.0 µg/L for microelements) and a digested real sample. Accuracy was evaluated as percentage recovery (R%) from spiked samples at appropriate fortification levels. Be, Sc, Rh, and Bi, not naturally present at detectable levels in pork meat, were used as internal standards to compensate for instrumental drift and matrix-related signal variations across the full mass range. Ultra-trace elements such as Li, Tl, Pb, and U, characterized by interference-free isotopes, were analyzed in no-gas mode, while He-KED mode was applied to the remaining elements to reduce spectral interferences. The analytical performance of the method is summarized in Table 3.

Instrumental LOQs ranged from 0.001 µg/L for Cd, Cs, Tl, and U to 15.8 µg/L for Ca. In some samples, concentrations of V, Cr, Ni, Ga, As, Ag, Tl, Pb, and U fell below their respective method LODs, with the proportion of sub-LOD values ranging from 0.2% (As) to 17% (Ga), confirming the adequate sensitivity of the method for trace and ultra-trace element determination in pork meat. In real samples, repeatability was below 10% for all elements except V, Ga, and Pb, which showed slightly higher values. Recovery ranged from 75 to 117% across all elements, indicating good accuracy.

Mineral concentrations were calculated on a dry matter basis and converted to a wet weight basis using the moisture content of each sample, according to Equation (2):

(2) $$C_{fresh}=C_{DM}\times \frac{1-{moisture}_{fresh}}{1-{moisture}_{DM}}$$

Raw concentration data were then normalized through defatted dry matter (DFDM) using Equation (3) and expressed as mg/kg DFDM or μg/kg DFDM.

(3) $$C_{DFDM}=C_{fresh}\times \frac{100}{DM\left(\%\right)-Fat(\%)}$$

Wet weight values allow direct comparison with existing literature and reflect the nutritional relevance of mineral intake by consumers. DFDM values eliminate the contribution of fat, which varied considerably among animals.

### 2.4. Statistical Analysis

Prior to data analysis, all variables were evaluated for normality and variance homogeneity. In this exploratory study, the sampling accounted for the variability arising from different pig breeds within farms and multiple slaughter sessions. Country-specific linear mixed-effects models (LMM) were attempted, with fixed effects including farm and slaughter age (as covariate), breed as a random effect nested within farm (samples from Poland), slaughter batch as a random effect nested within breed and farm (samples from Italy), and pairwise comparisons between estimated marginal means with the Bonferroni adjustment. LMM was not applicable to the Danish samples, which were obtained from a single farm and a single breed, and to the Spanish ones, which featured a structural confound between farm and breed and a constant slaughter age within each farm, preventing the estimation of these effects. Therefore, farm-level comparisons, considering the combined influence of husbandry system, diet, breed, and slaughter age, were used throughout the study, as LMM was unable to reliably and homogeneously disentangle the partial confounding that occurred between farm, breed, and slaughter age. Thus, when data normality and homogeneity of variance were met, analysis of variance (ANOVA) of isotope ratios and mineral elements (expressed on wet-basis data) was performed considering the farm as a fixed factor and using Tukey’s post hoc test to compare means for significant differences (*p* < 0.05). For normally distributed data with non-homogeneous variances, one-way ANOVA followed by Tamhane’s T2 post hoc test was used, whereas for non-normally distributed data, the Kruskal–Wallis test was applied, followed by the post hoc pairwise comparisons with Bonferroni correction when significant. Pooled SEM based on the pooled variance was reported.

The factor analysis with principal component extraction (PCA) was performed to explore multivariate patterns in isotope ratios and mineral elements (expressed on DFDM). All statistics were obtained by SPSS ver. 30.0 (SPSS Inc., Chicago, IL, USA).

## 3. Results and Discussion

The study provides valuable information on the stable isotope ratios of bio-elements (O, H, C, N, and S) and mineral elements determined in over 600 pork LTL muscle samples from pigs raised on 12 European farms across four countries (Denmark, Poland, Italy, and Spain). These farms differed in climatic and geographical conditions, pig breeds, feeding practices, and slaughter period (Table 1). The large sample size and variability in farming conditions make this dataset a useful reference for pigs from European countries with well-established breeding traditions.

Data on stable isotope ratios and mineral elements collected during the survey, covering both extensive and intensive farms, are reported in Tables S1–S6 in the Supplementary Materials, organized by country, farm, breed, and slaughter period. Specifically, Table S1 refers to samples from Denmark (intensive system, across different seasons), Table S2 to samples from Poland (intensive system), Table S3 to samples from Italy (extensive system), Table S4 to samples from Italy (intensive system, across different seasons), Table S5 to samples from Spain (extensive system), and Table S6 to samples from Spain (intensive system). The extensive and intensive systems involved in the study differed across most farming conditions, precluding direct comparisons of stable isotope ratios and mineral elements between the two systems. One exception is farm S1/s1 (Table 1), where animals of the same crossbreed were reared under both extensive and intensive conditions, but received different basal diets and were slaughtered at different ages and seasons.

### 3.1. Stable Isotope Composition

#### 3.1.1. Influence of the Country of Provenance

**Figure 1:**
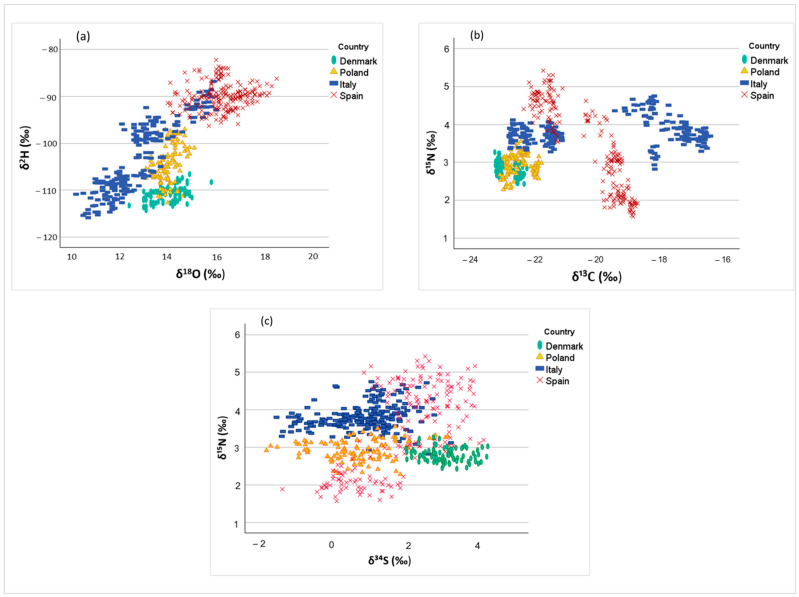
(**a**–**c**) Scatter plot of δ^18^O versus δ^2^H (**a**), δ^13^C versus δ^15^N (**b**), and δ^34^S versus δ^15^N (**c**) in defatted dry matter (DFDM) of pork *longissimus thoracis et lumborum* (LTL) samples from different geographical areas.

Stable isotope data from pork LTL samples are presented in Table 4, averaged by country of origin. To explore isotopic variability and clustering patterns, two-dimensional scatterplots of δ^18^O vs. δ^2^H (Figure 1a), δ^13^C vs. δ^15^N (Figure 1b), and δ^34^S vs. δ^15^N (Figure 1c) were displayed.

Beyond the trends suggested by the mean values in Table 4, the scatterplots in Figure 1 reveal a wide dispersion of samples, reflecting both overlap among countries and considerable within-country variability. Samples from the Spanish farms, located at the southernmost latitudes, generally showed higher average δ^18^O and δ^2^H values compared to those from countries at higher latitudes. Italian samples were broadly scattered across the plot, spanning a wide range of both δ^18^O and δ^2^H values (Figure 1a) and overlapping with some Spanish and Polish samples; the latter also overlapped with Danish samples. This pattern is consistent with Italy’s elongated geography and with the sampling strategy, which included farms in markedly different environments: one farm in central Italy near the coast, two in the Po Valley, and one in the pre-Alpine area further north.

The European samples analyzed in this study exhibited δ^18^O and δ^2^H values generally higher than those reported for pork from several regions of China [17,41], where values ranged from −105 to −123‰ for δ^2^H and from 5 to 10‰ for δ^18^O. Shin et al. (2018) [27] reported more negative δ^2^H values for Korean continental samples (−110 to −120‰), while Canadian samples showed even lower δ^2^H (−158 to −163‰) and δ^18^O (6.2–8.1‰) values, reflecting the influence of cold environmental temperatures, which reduce the abundance of heavy isotopes in precipitation and, consequently, in animal tissues [26]. Values reported by other authors [23,24] are not directly comparable with those of the present study, as they were determined on water extracted from meat by cryogenic distillation, whereas our analyses were performed on defatted freeze-dried matter.

Since the isotopic composition of precipitation becomes depleted in heavy isotopes with decreasing air temperature and correlates with that of drinking water [41], ambient temperature is a primary driver of δ^18^O and δ^2^H values in meat. These isotopes can therefore serve as environmental and geographical markers. The enrichment in heavy isotopes observed in Spanish samples is consistent with the warmer climatic conditions prevailing in Spain compared to Denmark, Poland, and central–northern Italy. It should be noted that drinking water isotopic data were not collected at the farm level in this study; the observed δ^18^O and δ^2^H patterns in pork muscle are therefore interpreted in relation to regional precipitation isoscapes, which represent the most proximate available proxy for the isotopic composition of local water sources. Overall, the spatial distribution of samples displayed in Figure 1a suggests a relationship between farm location and δ^18^O–δ^2^H signatures, allowing discrimination of meat from areas differing in latitude-related parameters such as temperature [42,43,44]. However, the δ^2^H–δ^18^O relationship observed in this study (δ^2^H = 4.0 × δ^18^O − 156.6; R^2^ = 0.88) depends not only on the well-established covariation of δ^2^H and δ^18^O along the Global Meteoric Water Line [45] but could also be related to dietary inputs [46].

The δ^13^C value reflects differences primarily associated with feeding strategies. C_3_ plants exhibit lower δ^13^C values due to stronger discrimination against ^13^C during photosynthesis, whereas C_4_ plants are relatively enriched [47]. Accordingly, Gonzales-Martín et al. reported mean δ^13^C values of −22.85‰ for wheat (C_3_) and −11.18‰ for maize (C_4_) [30], and feeding systems that include maize generally increase δ^13^C values in animal tissues [16,42].

Samples from Denmark and Poland, where diets are predominantly based on C_3_ cereals, clustered at more negative δ^13^C values (Figure 1b), showing a relatively homogeneous distribution consistent with similar husbandry practices across these countries (Table 1). In contrast, samples from Spain and Italy showed higher mean δ^13^C values but a wide dispersion in the δ^13^C vs. δ^15^N plot (Figure 1b), forming clearly distinct clusters in isotopic space. This reflects the heterogeneous feeding strategies within these countries, corresponding to the markedly different husbandry systems studied, ranging from free-range to intensive. Indeed, the Spanish production systems represent the most extreme contrast, particularly with respect to free-range finished Iberian pigs.

A subgroup of Spanish and Italian samples clustered at lower δ^13^C values, consistent with diets predominantly based on C_3_ plants. Conversely, another subset of Italian samples showed markedly enriched δ^13^C signatures, indicating mixed C_3_–C_4_ feeding regimes. A subgroup of Spanish samples occupied a low-to-intermediate δ^13^C range, which may reflect the presence of some maize in the diet or the consumption of acorns (Table 1), a C_3_ plant that exhibits higher δ^13^C values than other C_3_ plants [30].

Samples from Italy and Spain generally exhibited higher mean δ^15^N values than those from Denmark and Poland. The nitrogen isotopic composition of animal tissues is primarily associated with dietary nitrogen sources and soil management practices. Leguminous plants typically exhibit δ^15^N values close to that of atmospheric nitrogen (~0‰), while differences in fertilization strategies and nitrogen cycling can lead to enrichment in animal products [13,48,49]. Danish and Polish samples clustered tightly at lower δ^15^N values, while Italian samples displayed intermediate-to-high values and Spanish samples showed greater dispersion along the δ^15^N axis, reflecting more heterogeneous feeding and production systems.

Sulfur isotope ratios (δ^34^S) largely reflect feed origin, which is primarily linked to the local geology of crop-producing areas and, in coastal regions, to marine sulfate inputs from sea-spray deposition [5,50]. Danish samples fell within the range of 2–4‰ and were distinguishable from Italian and Polish samples, which also included negative values, whereas Spanish samples showed high variability, though predominantly positive values (Figure 1c). The variability observed in δ^34^S may be explained by a stronger dependence on the geology of feed-sourcing areas rather than on farm location itself. 

#### 3.1.2. Effect of Farm’s Husbandry Conditions

To better interpret the observed isotopic distribution, a farm-level comparison was performed for each country of sample origin, except Denmark, where samples came from a single farm. The results are reported in Table 5, Table 6 and Table 7 for Poland, Italy, and Spain, respectively.

Polish farms included in the study were managed under an intensive production system. Animals of different breeds were slaughtered at varying ages and fed diets with slightly varying composition, though all based on C_3_ plants such as barley, wheat, and rye. Despite this, samples from Poland showed significant differences in δ^2^H and δ^18^O between farms. While these parameters primarily reflect local rainwater composition, the observed differences cannot be explained by drinking water alone, given the similar latitude and altitude of the farms. The variability may therefore also be influenced by other factors, such as specific dietary components, breed, and slaughter period. The δ^13^C values of pork samples were higher in samples from farm p2, possibly reflecting a different isotopic contribution of the plant species included in the basal diet, although the differences were relatively small. δ^15^N values were higher in samples from farm p3, where the native Puławska breed was slaughtered at an older age than the crossbreeds raised on the other farms (Table 1). Since isotopic fractionation from dietary nitrogen sources can favor the accumulation of heavier isotopes over the animal’s lifetime [49,51], greater age at slaughter may contribute to elevated δ^15^N values. Sulfur isotopic composition also differed among the three Polish farms, likely reflecting distinct geological characteristics of feed-sourcing areas or differences in dietary inputs. However, since in this study the isotopic composition of the feed was not directly measured, the relationship between δ^13^C, δ^15^N, and δ^34^S values and dietary inputs cannot be clearly established. In addition, feed ingredients may be sourced from geographically distinct regions not related to the farm location.

Italian farms showed substantial diversity in their management systems. In addition to being located in geographically distant regions with distinct climatic characteristics, they adopted different production systems: semi-extensive (I1 and I2), intensive (i3), and intensive organic with outdoor access (i4). Feeding practices also varied, with some farms relying exclusively on C_3_ plants (i4) and others using a mix of C_3_ and C_4_ plants (I1, I2, i3). The breeds reared were either local (MR and CS with crossbreeds), which require longer growth periods, or commercial hybrids, slaughtered at an extended age for the production of typical PDO and PGI products. The above differences in management help explain the variations observed in Table 3. Differences in δ^2^H and δ^18^O values are consistent with the geographical locations of the farms. Farm I2, which showed the higher δ^2^H and δ^18^O values, is situated at the southernmost latitude among the Italian farms included in this study, and is close to the coast (approximately 40 km away). Conversely, i4 exhibited the lowest δ^2^H and δ^18^O values and is located further north, at a higher altitude near the Alps. Farms I2 and i3, located in the Po Valley in close proximity to each other, exhibited intermediate values. These differences in δ^2^H and δ^18^O values among Italian farms mainly reflect the different latitude, sea distance, and altitude, and are in accordance with the spatial distribution of δ^18^O of precipitation in Italy [52]. The δ^2^H and δ^18^O values of farm I2 are consistent with those reported for Sicilian pork samples analyzed under similar analytical procedures [31], which showed mean δ^18^O and δ^2^H values ranging from 14.3 to 15.6‰ and −86 to −78‰, respectively. The higher δ^2^H values observed in ‘Nero dei Nebrodi’ pigs from Sicilian farms compared to I2 reflect the environmental conditions associated with their more southerly location, likely enhanced by evapotranspiration in pasture plants. This process enriches δ^2^H and δ^18^O values in plant tissues and is more pronounced at higher temperatures, diminishing as temperatures decrease [53]. This effect is particularly evident in farm i4, where feed is produced on-site.

The location of farm i4 results in generally lower temperatures compared to the other Italian farms. Being situated far from the sea, it also receives precipitation depleted in ^18^O and ^2^H as heavy isotopes are progressively removed from vapor masses moving inland, rendering rainfall isotopically lighter with more negative δ^18^O and δ^2^H values [54]. Lower δ^18^O and δ^2^H values have similarly been reported in Chinese organic pork relative to conventionally produced samples [17]. A dilution effect from non-locally sourced feed cannot be excluded, given that the isotopic composition of hydrogen and oxygen in animal tissues is influenced by both feed and drinking water [44,55].

The δ^13^C values reflect the feeding regimens adopted and discriminate between farms. Farms I1, I2, and i3, which include maize in the diet (mixed C_3_–C_4_-based), showed values between −17 and −18.5‰, whereas the organic farm i4, relying exclusively on C_3_ plants such as barley and wheat, exhibited lower values (−21.9‰), consistent with those observed in Denmark and Poland (Table 3). Regarding δ^15^N, farm I2 showed the highest values among the Italian farms. Farm I2 raises local native breeds slaughtered at a later age than the other Italian farms. Enrichment in the heavier nitrogen isotope (^15^N) in animal muscle is driven by metabolic fractionation [49] and increases over time as dietary nitrogen is assimilated [51]; accordingly, as previously postulated for the Polish sample, older slaughter age may be associated with higher δ^15^N values, possibly reflecting cumulative dietary nitrogen exposure over time. However, diet and soil fertilization may also contribute to the observed isotopic variation.

Differences in δ^34^S were observed among farms located in Italian regions. The δ^34^S values in animal tissues can reflect the isotopic composition of feed [13], which is not locally sourced in most cases, except for farm i4, which produces its own feed. Findings from other livestock species (e.g., beef [16,42] and lamb [18]) suggest that such δ^34^S variations may be driven by dietary inputs.

In Spain, three farms (S1, S2, and s3) are located in the same geographic area, well known for the production of traditional Iberian pork products. These farms rear Iberian pigs or their crossbreeds outdoors in the Dehesa system (S1 and S2), slaughtering them at 14–23 months, while farm S1 operates under an intensive system with slaughter at 12 months. Farm s3 also rears Iberian pigs, consisting of sows slaughtered at a considerably older age than in conventional systems (50 months). Farm s4 differs from the others both in geographical location and in the use of a commercial hybrid breed slaughtered at a younger age.

The δ^18^O values showed variability among farms. Farm s1 exhibited the highest δ^18^O values, a pattern that may reflect a seasonal effect: s1 animals were slaughtered in May, a warmer month in this area, whereas animals from S1, S2, and s3 were slaughtered in winter. Indeed, δ^2^H and δ^18^O values can also reflect physiological effects, as higher temperatures enhance water turnover and evaporative water loss, leading to enrichment of heavy isotopes in body fluids and tissues [56]. Additionally, the older slaughter age of some animals may have contributed to a gradual enrichment in heavier isotopes in tissues over time. Farm s4, located near the sea, showed higher values than S1, S2, and s3. Farm s3, situated slightly further north, showed a lower δ^18^O value. These results are in agreement with the isoscape of the Iberian Peninsula [57].

Farms s1 and s4, fed mixed C_3_–C_4_ diets, showed the highest δ^13^C values among the Spanish farms, though slightly lower than those of the Italian farms, suggesting a lower proportion of maize in the diet. Conversely, farm s3, fed exclusively on C_3_ plants, showed the lowest δ^13^C values, comparable to those from Denmark, Poland, and Italian farm i4. Animals from S1 and S2, reared in the Dehesa on acorns and grasses, exhibited intermediate δ^13^C values, consistent with the δ^13^C signature of acorns (−21.0‰) [29]. The slightly lower δ^13^C in S2 may reflect the inclusion of C_3_-based compound feed. Differences in δ^15^N values were also observed among farms, likely reflecting the combined effect of diet, breed, and age at slaughter. Farm s4, with commercial hybrids reared for 7 months under an intensive C_3_ diet, exhibited the lowest δ^15^N values. In contrast, pure Iberian or Iberian × Duroc pigs from the other farms were reared for longer periods (12–50 months) on C_3_ or C_3_ with acorn diets, showing higher δ^15^N values, consistent with a possible enrichment in the heavier nitrogen isotope in muscle tissue mediated by cumulative dietary exposure. However, it seems that the increased δ^15^N values remain constant at increasingly high slaughter ages (e.g., pigs slaughtered when 23 and 50 months old), suggesting that no further enrichment occurs at very advanced pig age. The δ^34^S values were lower in samples from farm s4 than in those from other Spanish farms, despite its proximity to the sea. These differences likely reflect a combination of environmental characteristics (soil, geology, and atmosphere) of the feed-sourcing areas, as well as the distinct breed and slaughter age of the animals. Previous research has shown that δ^34^S can discriminate between Iberian pigs fed acorns and those fed compound feed [30]; however, in the present study, no clear δ^34^S trends were observed among different feeding regimens, despite S1 and S2 farms relying largely on acorn-based diets. To differentiate between these two very similar diets, volatilome analysis by gas chromatography coupled with ion mobility spectrometry of fecal samples has been proposed [58,59].

#### 3.1.3. Influence of Slaughter Season

**Figure 2:**
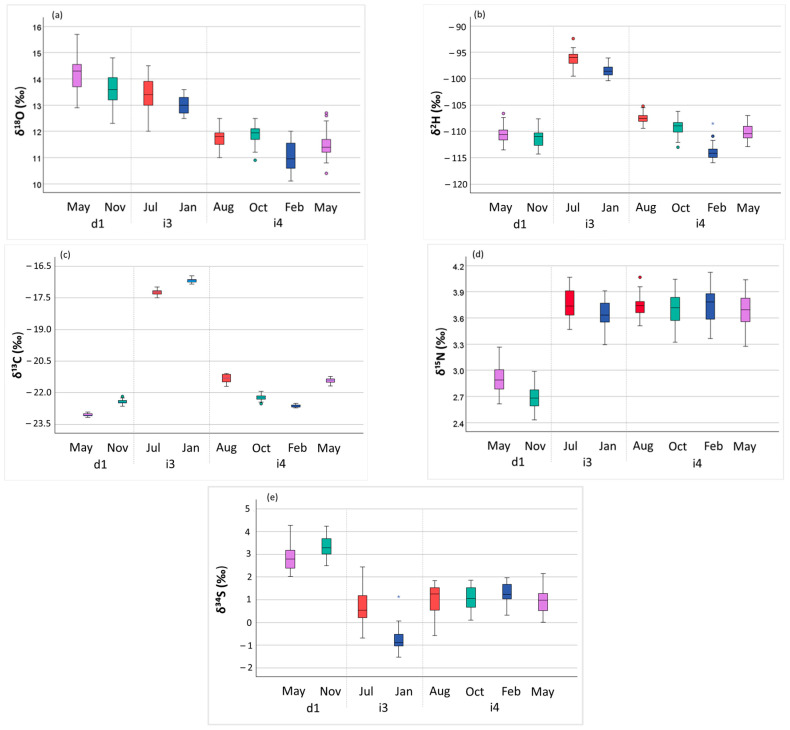
(**a**–**e**) Stable isotope ratios (δ^18^O, δ^2^H, δ^13^C, δ^15^N, and δ^34^S, ‰) measured in DFDM of LTL pork samples from three farms (d1, i3, and i4) collected at different times throughout the year. Boxplots represent the distribution of isotopic values for each sampling period within each farm. Outliers are indicated by an asterisk (*).

Previous studies have demonstrated that slaughter season can influence isotope ratios variability in animal body fluids and tissues [56,60]. In this study, a subset of LTL samples was obtained from pigs raised under the same husbandry conditions within each farm of origin (d1, i3, and i4), but slaughtered at different times of the year (Table 1). Seasonal variability of δ^18^O, δ^2^H, δ^13^C, δ^15^N and δ^34^S is shown in Figure 2a–e.

Samples collected in winter and late autumn exhibited, on average, lower δ^2^H and δ^18^O values compared to those obtained in summer and late spring (Figure 2a,b). This effect was more pronounced for δ^2^H in farm i4, where δ^2^H values shifted from −107‰ in samples obtained in August to −114‰ in February. This trend can be attributed to winter air cooling, during which heavy isotopes are preferentially retained in the liquid or solid phase, resulting in isotopically lighter precipitation. Consequently, in winter, rainwater is isotopically lighter than in warmer summer periods, and δ^18^O and/or δ^2^H values reflect these changes. In summer, δ^2^H and δ^18^O values in body fluids are further elevated by greater water loss through respiration and skin evaporation, as well as changes in the ratio between ingested and excreted water [56]. A similar seasonal pattern has been reported for beef tissues over approximately 18 months [60], with enrichment in heavy isotopes (δ^2^H and δ^18^O) during the warm season (spring–autumn) and values approaching those of drinking water in winter. A comparable seasonal trend was observed in farm i3, where pigs were slaughtered in July and January, while no differences were detected in farm d1, which slaughtered animals in May and November, months with relatively similar temperatures.

The variation in δ^13^C values observed across different slaughter periods indicates the presence of a seasonal effect, although no consistent pattern emerged across farms. A previous study reported lower δ^13^C values in winter than in summer [17]. In the present study, farm i4 showed higher δ^13^C values during the warmer months, whereas the opposite trend was observed in farms i3 and d1 (Figure 2c). Overall, these results suggest that, while a seasonal effect exists, δ^13^C fluctuations may also be driven by farm-specific feeding strategies and changes in feed composition throughout the year. The limited within-farm variation in δ^15^N values indicates that this parameter is relatively stable and less sensitive to seasonality or short-term dietary changes (Figure 2d). Regarding δ^34^S, opposite seasonal trends were observed in farms d1 and i3: higher values were recorded in late autumn for d1 and in summer for i3 (Figure 2e). These contrasting results suggest that dietary inputs, rather than seasonality alone, may drive variation in the δ^34^S signature.

### 3.2. Mineral Elements Profile

The mineral elements identified in pork LTL are presented in Table 4, Table 5, Table 6 and Table 7 and were classified into three groups:

- Macronutrients (K, P, Na, Mg, and Ca), present at high concentrations, and essential for growth, skeletal development, neuromuscular function, and osmotic and acid–base balance [61];
- Micronutrients (Zn, Fe, Cu, Se, Mn, Cr, Mo, Ni, and Co), essential minor elements required in small amounts for critical biological functions;
- Non-essential elements (Rb, Sr, Cs, Ba, Pb, Ag, Li, As, Cd, V, Tl, Ga, and U), including micro- and trace elements without a recognized biological role in animals, primarily reflecting environmental or soil-derived sources.

As reported by several authors, the amounts of mineral elements in LTL are strictly related to feeding practices [12,25], including the use of mineral supplements in the diet [5]. Among macronutrients, K and P were present at the highest concentrations in pork LTL, followed by Na and Mg. These elements play essential roles in pigs, supporting growth, neuromuscular function, and the maintenance of osmotic and acid–base balance [61]. Calcium (Ca), primarily associated with bone tissue, showed the lowest concentration in the LTL. Among micronutrients, Zn and Fe were the most abundant elements, reflecting their key roles in oxygen transport, metabolism, and overall development of the animal. Other essential mineral elements—Cu, Se, Mn, Cr, Mo, Ni, and Co—were detected at lower concentrations and contribute to a range of physiological processes, including growth and tissue development, enzymatic reactions (Mo and Ni), antioxidant defense (Cu, Se, and Mn), metabolism (Cr and Co), and immune function (Se) [62]. Selenium concentrations in animal tissues largely reflect its content in consumed plants, but are also strongly influenced by supplementation practices, as Se is commonly added to pig diets to meet nutritional requirements [19].

Among non-essential minerals, Rb was the most abundant. It is naturally prevalent in soil and readily taken up by plants due to its ionic similarity to K^+^, and may therefore accumulate in pigs through feed intake depending on dietary composition [19,63]. The remaining non-essential elements were detected at trace (Sr and Cs) and ultra-trace levels (Ba, Pb, Ag, Li, As, Cd, V, Tl, Ga, and U). These elements may originate from anthropogenic contamination or from natural phenomena such as soil erosion [5], and are therefore linked to the specific geology of the production area. In particular, Rb, Sr, Cs, Ba, Li, and Ga are primarily associated with the natural geology of soils, whereas Pb, Ag, As, Cd, V, Tl, and U are more commonly linked to anthropogenic sources, although local geology may also contribute to their presence. Lead (Pb) and cadmium (Cd) are classified as metal contaminants in pork meat under Commission Regulation (EU) 2023/915 [64]; in this study (Table 4, Table 5, Table 6 and Table 7), both were consistently detected at concentrations well below the legal limits for meat (0.10 mg/kg fresh weight for Pb and 0.050 mg/kg fresh weight for Cd). Other potentially toxic elements not currently regulated, such as As, Tl, and U, were detected only at ultra-trace levels (<1.0 µg/kg fresh weight).

#### 3.2.1. Influence of the Country of Provenance

Mineral concentrations in pork LTL, expressed on a wet weight basis, are reported by country of origin in Table 4, revealing a distinct mineral fingerprint for each country. The results are in line with recent literature [23,24,25,31,32]. Overall, elemental concentrations were broadly comparable across the four countries; however, the relatively large standard deviations indicate substantial within-country variability, likely reflecting differences in farming practices among individual farms, including feeding strategies, supplementation protocols, and herd management conditions. It should be noted that detailed feed ingredient composition and mineral supplementation levels were not available for most farms due to commercial confidentiality, and no elemental analysis of feed was performed. The interpretation of mineral patterns is therefore based solely on meat data, as direct attribution to specific dietary or environmental sources was not possible.

Some elements seem to characterize the mineral profile of specific countries: Zn and Se were higher in Italian and Spanish samples, while Fe was notably higher in Spanish pork. Iron and zinc metabolism are closely associated with the physiological demands of growing animals. Animals slaughtered at older ages exhibit higher Fe and Zn concentrations in muscle than younger ones [65,66]; since pigs from Italy and Spain were typically slaughtered at older ages than those from Poland and Denmark (Table 1), this may account for the higher Fe and Zn levels observed in their meat. The elevated concentrations of Zn and Fe in Italian and Spanish pigs are of particular interest, as most of these animals are destined for PDO dry-cured ham production [67]. Both elements are considered important quality attributes, as they are involved in oxidative reactions and in the formation and stability of the red color, particularly in dry-cured meat products [68]. Specifically, both Fe and Zn play a role in the formation of zinc protoporphyrin IX, a natural red pigment that contributes to stable color development in long-aged, nitrite-free, dry-cured meats, with total iron content and zinc levels being positively associated with its formation. Selenium concentrations in animal tissues largely reflect the Se content of feed plants, but are also strongly influenced by supplementation practices, as Se is commonly added to pig diets to meet nutritional requirements [19]. Danish meat showed elevated concentrations of certain non-essential elements, including Sr, Cs, and Ag, whereas Polish samples were characterized by higher levels of Cr, Tl, and Pb.

#### 3.2.2. Effect of Farm’s Husbandry Conditions

Table 5, Table 6 and Table 7 report the mineral composition of pork LTL samples from farms in Poland, Italy, and Spain, respectively, enabling intra-country comparisons among farms.

In Poland, three intensive farming systems were compared (Table 1). Farm p1 generally exhibited lower concentrations of mineral elements, including macronutrients (K, P, Na, Mg, and Ca), micronutrients (Fe, Cu, and Mn), and non-essential elements (Rb, Cr, Ni, As, V, Tl, Ga, and U). Farm p2, in contrast, showed higher concentrations of several micronutrients (Cr, Mo, Ni, and Co) and non-essential elements (Ba and Ag). The mineral profile of farm p3 was broadly similar to that of farm p2, with the exception of certain non-essential elements (Ba, Cs, Pb, As, Cd, and Ga). Differences in macro- and micronutrient concentrations likely reflect variations in dietary formulation, particularly in the mineral supply of the feed. The higher levels of Zn, Cs, Pb, As, and Cd observed in farm p3 may be related to the older slaughter age of the Puławska breed, as local breeds grow slowly and these elements are known to accumulate in animal tissues over time (Table 5).

The Italian farms showed distinct mineral profiles (Table 6). Among macronutrients, farms I2 and i3 were characterized by higher K, Mg, and P concentrations than I1, while i4 was distinguished by relatively lower P and Ca levels. Regarding micronutrients, Fe and Zn concentrations were generally elevated in I1, I2, and i3. Farm I2, which reared Cinta Senese pigs slaughtered at 13–14 months, showed the highest Fe level. Farm I1 raised both a local breed, Mora Romagnola, slaughtered at 14 months, and a commercial LW × D crossbreed reared until 10 months. Notably, Fe concentrations in Mora Romagnola LTL reached 5.06 mg/kg, that exceed even the average value recorded for the Cinta Senese group (Table S3, Supplementary Materials). These findings support the role of breed-specific characteristics—including genetics, slaughter age, and weight, and physical activity—in influencing Fe accumulation in muscle tissue [66,69,70]. Farm I2 also exhibited higher Se concentrations, while Mo levels were lower in i3, differences that may reflect variations in feed composition and supplementation strategies.

Cobalt concentrations were particularly notable in farm I2, where pure Cinta Senese pigs showed higher levels (2.16 mg/kg) compared with CS × LW and CS × D crossbreeds (1.20 and 1.26 mg/kg, respectively) (Table S3, Supplementary Materials) and with other farms. This suggests that Co accumulation may be influenced by the animal’s genetic background. In mammals, cobalt is biologically relevant primarily as a component of vitamin B_12_, so tissue Co levels reflect differences in B_12_ utilization and retention rather than direct dietary intake [71]. Traditional local breeds such as Cinta Senese are characterized by a muscle phenotype oriented towards oxidative metabolism, which entails higher requirements for mitochondrial cofactors and likely contributes to the elevated tissue-bound Co levels observed in these breeds [72,73,74].

The organic farm (i4) showed generally lower mineral concentrations, probably associated with reduced mineral supplementation and the use of feed ingredients with distinct mineral compositions. Furthermore, Pb, V, Tl, and U concentrations at farm i4 were the lowest among all farms in the study. This may be attributable to the on-farm production of feed in an area remote from major sources of environmental pollution. Since Pb accumulation in meat largely reflects soil Pb levels, which are strongly influenced by atmospheric deposition and air pollution, the low Pb concentrations observed may indicate limited environmental contamination in the production area [75].

Among the Spanish samples, farm s4 displayed a distinct mineral profile compared with the other farms, as its production system differed most markedly, involving intensively reared commercial breeds (Table 7). The elevated concentrations of K, P, Mg, Se, and Mn at this farm may indicate substantial use of mineral supplementation in the diet, while the lower Fe, Zn, Cu, Cr, and As levels are likely associated with the younger slaughter age (7 months) relative to the other groups. Farms S1 and S2, where pigs are reared in the Dehesa under free-ranging conditions and fed primarily on acorns and natural pasture (with additional feed supplementation in S2), exhibited a mineral profile distinct from that of intensively reared animals. In particular, macronutrients such as K, P, and Mg, as well as the micronutrient Se, were present at lower concentrations than in intensive systems, likely reflecting the limited use of mineral supplementation in these extensive production systems. However, chromium concentrations were higher in S2 than in the intensive farms, a difference that may be linked to the grazing environment or specific supplementation practices. Supplementation with organic chromium in diets for growing and finishing pigs has been reported to increase carcass muscle percentage while reducing fat deposition, and in heavy pigs, it has been shown to improve feed efficiency, average daily gain, and lean cut yield [76].

Farm s3, operating under an intensive system with purebred Iberian pigs slaughtered at approximately 50 months of age, showed elevated concentrations of several elements, particularly Fe and Zn (8.37 and 23.6 mg/kg, respectively), which are strongly associated with breed and slaughter age. Similarly high Fe and Zn levels were observed in farms S1 and S2, where purebred Iberian and Iberian crossbreed pigs were reared for longer periods (14–23 months). Farm S1, managed extensively under the Montanera system, exhibited mineral profiles consistent with previously reported high Fe contents in Iberian pigs. Overall, the enrichment of Zn, Fe, and Cu observed in Spanish Farms S1, S2, and s3 is consistent with published data for native pig breeds, which are characterized by a more oxidative muscle metabolism and greater adaptability to environmental stress [77,78]. These elements act as cofactors of antioxidant and metabolic enzymes (e.g., superoxide dismutase, glutathione peroxidase, and cytochromes), suggesting a greater functional endowment of muscle tissue. In particular, higher Fe levels may reflect increased myoglobin content, as commonly reported in local breeds compared to commercial ones, possibly influenced by the foraging activity of the former [79]. Similarly, higher Zn and Cu concentrations may reflect greater expression of metalloproteins and metallothioneins, which are involved in both essential metal homeostasis and cellular defense mechanisms. Furthermore, animals reared under extensive conditions are generally exposed to greater environmental variability (e.g., temperature fluctuations, physical activity, and pathogen exposure), which may stimulate endogenous antioxidant defenses. Since Zn and Cu are key components of the Cu/Zn superoxide dismutase enzyme, increased antioxidant demand could favor enhanced retention of these elements in muscle tissue, partially explaining the higher Zn and Cu concentrations detected in the LTL of extensively reared Spanish pigs [41].

Selenium content was higher in intensive farms both in Spain (s4) and in Italy (i3). Selenium levels in meat can reflect both the mineral supplements administered to the animal and the Se content of cereals grown in Se-rich soils, though distinguishing between these two contributions is difficult [5]. Higher Se concentrations in conventionally reared pork compared to organically reared pork have already been reported in the literature, suggesting that Se may be a useful marker to discriminate animals reared under different production systems within the same geographical origin [17].

In pork samples from Spain and Italy, Rb was significantly higher in intensively reared animals, a finding likely reflecting diets containing Rb-rich plants or feed supplemented with this mineral. In addition, Rb and Cs—elements that mimic potassium—and Li, V, and Cr—often regarded as metabolic tracers rather than essential nutrients—were higher in commercial pigs. This pattern likely reflects a more dynamic and less selective ion metabolism characteristic of genotypes selected for high production performance, rather than a specific physiological adaptation.

An interesting feature is the positive correlation between As, Fe, and Zn, which can be attributed to a combination of nutritional, environmental, and biochemical factors. Fe and Zn are commonly present in feed as essential elements, whereas arsenic can be introduced through feed, water, or soil contamination [5]. Long-term exposure to these sources may promote concomitant absorption and accumulation of these elements in muscle tissue, thus explaining the correlations observed in our samples. Indeed, these elements are largely associated with proteins through binding mechanisms involving metalloproteins. Zn and Fe are primarily bound to metalloproteins and metallothioneins, whereas arsenic, particularly in its trivalent form, has a high affinity for the thiol groups of cysteine residues within these proteins [80]. Consequently, tissues with higher protein content and elevated metallothionein expression may promote the concomitant retention of Zn and As, with Fe being indirectly associated through both heme and non-heme proteins.

#### 3.2.3. Effect of Slaughter Season

Although research specifically addressing the effect of slaughter season on mineral profiles in pig tissues is limited, studies on pork quality indicate that seasonal factors can influence muscle composition and chemical characteristics [81], suggesting that mineral content may also be affected. In this study, the seasonal effect was evaluated in samples from farms d1, i3, and i4, where pigs were raised under the same husbandry conditions but slaughtered at different times of the year. Principal component analysis (PCA) was performed on the mineral composition data to identify patterns and highlight potential seasonal variation in the mineral profiles of the samples.

In Danish samples, pigs slaughtered in May showed higher concentrations of macronutrients (Ca, Na, Mg, K, and P), selenium, and arsenic than those slaughtered in November (Table S1, Supplementary materials). Similarly, samples from Italian farm i3 slaughtered in summer were characterized by higher concentrations of Ca, Mg, Na, Fe, and Co, whereas those slaughtered in winter showed the highest levels of Se, Rb, Cs, and Tl. Samples from farm i4, fed organic feed, also differed between slaughter seasons in Se, Rb, Cs, and Tl, though in a different order than i3 (Table S4, Supplementary materials).

The observed seasonal differences in muscle mineral composition are likely attributable to environmentally driven physiological adaptations, particularly those related to summer heat stress. High ambient temperatures may induce changes in feed intake, water consumption, acid-base balance, and electrolyte homeostasis in pigs, leading to alterations in the distribution and retention of major minerals [82,83]. Under heat stress conditions, increased water intake and respiratory activity, together with modifications in mineral metabolism, may contribute to higher muscle concentrations of Ca, Mg, Na, K, and P in animals slaughtered during summer [84]. Conversely, pigs slaughtered in winter showed higher concentrations of trace elements such as Se, As, Rb, and Cs, which are characterized by slower metabolic turnover and weaker homeostatic regulation than major electrolytes. In the absence of heat stress, metabolic conditions may favor greater tissue retention and accumulation of trace elements, whereas during summer, an increased utilization of antioxidant systems and higher excretion rates may reduce their muscle deposition, particularly for selenium, which plays a key role in the antioxidant response to thermal stress [83].

Overall, these results suggest that season represents a relevant source of variation in the mineral composition of pork, even under a constant diet. Moreover, it should be noted that animals slaughtered in winter spent most of the growing period during summer, whereas pigs slaughtered in summer were mainly reared under colder conditions. Therefore, seasonal differences in mineral composition may reflect both short-term pre-slaughter effects, particularly for major electrolytes, and long-term cumulative effects during growth, especially for trace elements characterized by slower metabolic turnover. Although the diet was theoretically identical throughout the rearing period, seasonal variations in feed supply and different batches cannot be completely ruled out. In commercial feed production, changes in ingredient sourcing, storage duration, and environmental conditions between summer and winter may result in slight variations in mineral and trace element composition, even for the same feed formulation. Such an effect has been reported especially for trace elements, whose concentrations are strongly influenced by the mineral profile of feed ingredients and premixes. Seasonal variability in feed composition may have contributed, at least in part, to the differences observed in muscle mineral content.

**Figure 3:**
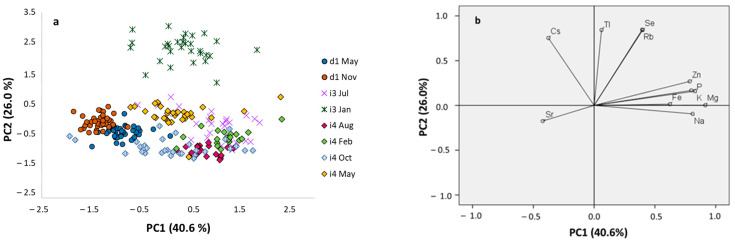
Principal component analysis of mineral elements. (**a**) Score plot of LTL pork samples clustered by farm and slaughter period (PC1 vs. PC2). (**b**) Rotated loading plot of mineral elements. Vector direction shows correlation, and length indicates each variable’s contribution to the components.

To better evaluate the effect of slaughter period on the mineral profile of pork meat, principal component analysis (PCA) was performed on the full mineral dataset; elements with the highest loadings were subsequently selected (Mg, Na, K, P, Fe, Zn, Se, Rb, Sr, Cs, and Tl). The first two principal components explained 66% of the total variance, with PC1 accounting for 40% and PC2 for 26% (Table S7 Supplementary Materials). Major elements such as Mg, Na, K, P, Fe, Zn, together with Sr, were loaded predominantly on PC1, whereas Se, Rb, Cs, and Tl were mainly associated with PC2. In the score plot (Figure 3), most nutritional elements clustered in the first quadrant, with Se and Rb closely associated, as well as Fe, Zn, P, K, Mg, and Na. These elements are commonly linked to dietary supplementation and are therefore strongly influenced by feeding strategies and husbandry practices. In contrast, Sr and Cs, typically reflecting environmental background, were located in the third and the fourth quadrants, respectively.

Grouping of samples from the same farm by slaughter season can be identified in the PCA space. Samples from farm d1, although located within the same quadrant (Q3), were clearly separated, suggesting that slaughter season may influence the mineral fingerprint. For farm i3, the winter group (i3-Jan) was positioned in the upper part of the score plot, associated with Rb, Se, Tl, and Cs, and clearly separated from the summer group (i3-Jul). As Rb and Se are commonly associated with dietary intake, this separation may reflect differences in supplementation intensity throughout the year, with animals slaughtered in January potentially receiving higher levels than those slaughtered in July. For farm i4, separation was less pronounced but still evident: samples slaughtered in August and May were clearly differentiated along PC2, while those collected in February and October were separated along PC1. Overall, these findings indicate that slaughter season can influence pork mineral profiles within the same production system, likely through the combined effects of feed formulation, supplementation strategies, and environmental factors.

### 3.3. Multivariate Analysis of Stable Isotope Ratio and Mineral Elements Data

A preliminary PCA was conducted on the mineral composition of 612 samples grouped by farm of origin, to identify the elements contributing most to the variability of the main components (loadings > 0.5). Based on this analysis, a subset of minerals (Na, Mg, P, K, V, Mn, Fe, Zn, Ga, As, Se, Rb, and Cs) was selected. An exploratory PCA was then performed on the combined dataset of 612 samples, integrating five stable isotope ratios (δ^13^C, δ^15^N, δ^34^S, δ^18^O, and δ^2^H) with the previously selected mineral elements. The analysis enabled the evaluation of sample clustering and the investigation of relationships between isotopic ratios and mineral composition. Samples were coded by farm origin, based on evidence that farm-specific husbandry conditions—including feeding strategies, management practices, and local environmental factors—influence both isotopic signatures and elemental profiles.

**Figure 4:**
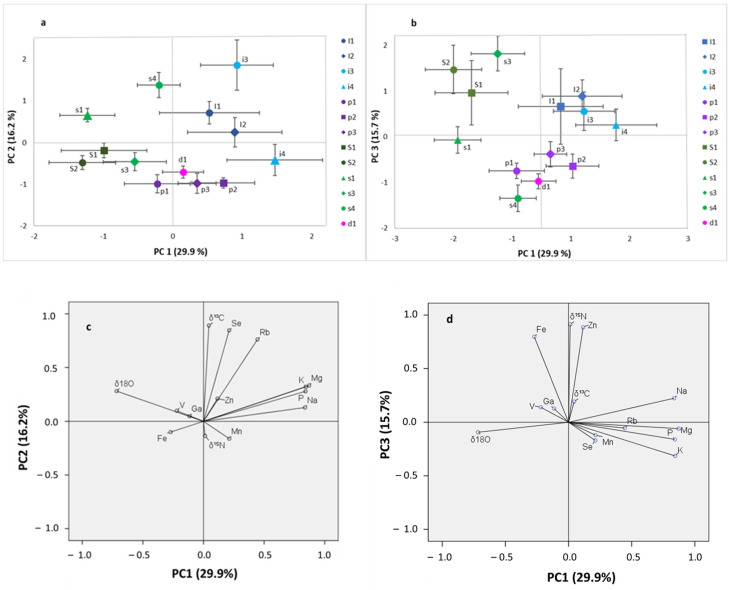
Principal component analysis of selected stable isotope ratios and mineral elements. Score plot of samples by farms, (**a**) PC1 vs. PC2, (**b**) PC1 vs. PC3. Rotated loading plots of isotope ratio and mineral elements, (**c**) PC1 vs. PC2, (**d**) PC1 vs. PC3. Vector direction shows correlation, and length reflects each variable’s contribution to the components.

The component matrix identified four principal components, accounting for 75% of the total variance (Table S8, Supplementary Materials). PC1 (30% of variance) was mainly driven by the δ^18^O and the macronutrients Mg, Na, K, and P, reflecting both a geographical signal, associated with δ^18^O, and farm-specific mineral supplementation practices. PC2 (16% of variance) was predominantly influenced by δ^13^C and the elements Se and Rb, linking this axis to dietary composition, specifically C_3_ versus mixed C_3_–C_4_ plant intake, as well as to specific microelements present in feed. PC3 (15% of variance) was characterized by Fe, Zn, and δ^15^N, variables positively associated with slaughter age and with each other (Pearson’s r: δ^15^N–Fe = 0.637, δ^15^N–Zn = 0.702, Fe–Zn = 0.659). PC4 (13% of variance) was mainly defined by trace elements such as V, Mn, and Ga, associated with environmental inputs. Although PC1 explained the largest proportion of variance, PC2, PC3, and PC4 provided complementary information on different production-related factors. PCA score plots based on the first three components are presented in Figure 4a,b, where samples are displayed as centroids of farm scores ± standard deviation (thin bars), given the large number of data points to be plotted.

Along PC1, samples were separated according to geographical origin (Figure 4a,b). Spanish farms showed negative scores, driven primarily by higher δ^18^O values, whereas Polish and Danish farms clustered at intermediate scores. Italian farms were positioned at positive scores, reflecting higher concentrations of macronutrients such as Mg, Na, K, and P. Separation along PC2 (Figure 4a) reflected dietary composition, with pigs fed cereal-based C_3_ diets located at lower scores and those receiving mixed C_3_–C_4_ diets at higher scores. Samples from animals fed exclusively C_3_-based diets (s3, i4, d1, and the Polish farms) were located at the lower end of PC2, with Farm S1—characterized by acorn-based feeding—occupying an intermediate position. Farms I2, I1, S1, s4, and i3 showed progressively more positive PC2 scores. In the case of S2, which uses both acorns and feed, the δ^13^C values (Table 5) place S2 samples lower than S1, which relies only on acorns. Among Italian farms, the high δ^13^C value of i3 (Table 5) explains its position along PC2, suggesting that the portion of maize in the feed is higher than in other farms.

PC3 (Figure 4b) mainly discriminated between breed type and slaughter age. The broad variability in slaughter ages across the dataset (Table 1) reflects the diversity of breeds, farming systems, and production purposes (fresh meat or processed products) represented in the study. Samples from pigs slaughtered at older ages (such as at 50 months in farm s3) highlight the effect of slaughter age on δ^15^N, Fe, and Zn, all of which show increasing values in farms where pigs are slaughtered later (Table 5). This information, derived from δ^15^N isotopic data and Fe and Zn concentrations in pork meat, is of great interest to PDO supply chains, where meeting minimum slaughter age requirements is mandatory for PDO eligibility. Conversely, negative PC3 scores were associated with samples from farms rearing fast-growing pigs slaughtered at younger ages (5–7 months).

Within similar production contexts, groups comprising pure local breeds tended to show higher PC3 values, reflecting both older slaughter ages and breed-specific muscle characteristics. For example, among Polish breeds and crossbreeds, farm p3 (Puławska) ranked highest along PC3. Within the Italian farms, local breeds were distributed at higher PC3 values compared to commercial crosses. Among the Spanish farms, even under intensive conditions, pure Iberian pigs consistently clustered at higher PC3 values relative to farms using commercial hybrid breeds.

Overall, the PCA highlights the complexity of European pig production systems, which encompass substantial variability in breed, slaughter age, feeding strategies, and mineral supplementation practices. These findings reinforce the need for a multivariate approach when addressing issues of authenticity, traceability, transparency of practices, and consistency with intended use.

Several limitations of the present study should be acknowledged. The hierarchical structure of the dataset (country > farm > breed > slaughter age > batch/season), in which breed, feeding strategy, and slaughter age were sometimes unique to individual farms, prevented the independent modeling of these factors and the assessment of their interactions. The dataset structure also prevented the application of supervised classification models. A Linear Discriminant Analysis was explored, but the resulting classification model likely appeared to capture farm-specific features, a model truly representative of pork origin at a country-level pattern. Thus, the development of validated classification models based on more balanced datasets, which take into account latitude, altitude, distance from the sea, husbandry system, breed, diet, and seasonality when selecting farms, remains an important future direction. Additionally, detailed feed composition and mineral supplementation data were not available for most farms, and no isotopic or elemental analysis of feed or drinking water was performed, preventing a direct attribution of the observed isotopic and elemental profiles to specific dietary or environmental sources.

## 4. Conclusions

The study showed that stable isotope ratios and mineral elements of pork of European origin are influenced by both geographic origin and farming practices. The obtained values of δ^2^H and δ^18^O, linked to geographic and feeding conditions through drinking water, differed among farms within the same country, reflecting variations primarily associated with farm location, diet, and slaughter period. The δ^13^C values were mainly associated with dietary composition, specifically the type and relative contribution of C_3_ and C_4_ plants. An increase in slaughter age was associated with higher δ^15^N, Fe, and Zn levels, possibly reflecting cumulative dietary exposure over time, although confounding effects of breed and production system should be considered. These parameters may be considered potential markers of breed, system, and pork meat from mature animals, destined for high-quality dry-cured products, with particular reference to PDO and PGI supply chains. Mineral composition was closely related to feed formulation and mineral supplementation strategies. Seasonal variability affected both isotopic and mineral parameters, likely due to changes in climatic conditions and feed availability throughout the year. Therefore, to fully document pork traceability and authenticity, information on geographical location and production system must be complemented by data on basal diet, supplementation strategies, breed, slaughter age, and period.

Finally, multivariate analysis highlighted the combined contribution of isotopic and elemental markers in clustering meat samples according to both geographical origin and rearing system. In particular, farms with similar management conditions—including feeding strategies, breed, and degree of extensiveness—tended to cluster together, while still showing distinctions between countries of origin. Although these findings are preliminary, they suggest that isotopes and minerals can provide a basis for the development of a European reference database. Expanding this database to include more European countries and a greater number of farms will be essential for developing and validating quantitative models applicable to authenticity assessment, safety verification, production standards compliance, and the protection of high-value supply chains against fraud, including breed substitution.

## Figures and Tables

**Table 1:** Overview of sample collection, reporting provenance of the pigs, farm ID, and GPS coordinates, farming system, diet type, breed/crossbreed or commercial line, slaughter age, and period.

Pig’s Provenance	Farm		Farming System	Photosynthetic Type of Diet	Breed ^2^	Slaughter	
	ID ^1^	GPS Coordinates				Age (Months)	Period
Denmark	d1	56.28° N, 9.36° E	Intensive	C_3_	DU × (LA × Y)	6	May and November 2023
Poland	p1	52.60° N, 16.50° E	Intensive	C_3_	LW	5.5	January 2024
					LW × PLW	5.7	
					PLW	6	
					PIC^®^	5	February 2023
	p2	52.95° N, 17.27° E	Intensive	C_3_	(LW × LA) × (DU × P)	5	May 2023
	p3	52.89° N, 16.65° E	Intensive	C_3_	PLW	7	October 2023
Italy	I1	44.22° N, 11.77° E	Semi-extensive	C_3_ and C_4_	LW × DU	10	June 2023
					MR	14	July 2023
	I2	42.76° N, 11.11° E	Semi-extensive	C_3_ and C_4_	CS	13–14	July 2023
					LW × CS		June 2023
					DU × CS		
	i3	44.64° N, 10.92° E	Intensive	C_3_ and C_4_	DU × LW	10	July 2023 and January 2024
	i4	44.38° N, 7.55° E	Intensive with outdoor access	C_3_ (organic)	Topigs^®^	10	August and October 2023, February and May 2024
Spain	S1	37.80° N, −5.02° W	Extensive Montanera	C_3_ (Grass, acorns)	IB	14	January 2024
					IB × DU		February 2024
	s1		Intensive	C_3_ and C_4_	IB × DU	12	May 2024
	S2	37.89° N, −4.78° W	Extensive Cebo de Campo	C_3_ (Grass, acorns, and feed)	IB	23	March 2023
					IB × DU		
	s3	38.49° N, −5.14° W	Intensive Outdoor	C_3_	IB	50	January 2024
	s4	37.67° N, −1.69° W	Intensive	C_3_ and C_4_	LW × LA	7	February 2024

^1^ Farms are identified by an alphanumeric code indicating country and farm number. Capital and lowercase letters indicate extensive/semi-extensive and intensive systems, respectively. ^2^ LW = Large White; LA = Landrace; DU = Duroc; IB = Iberian; CS = Cinta Senese; MR = Mora Romagnola; PLW = Pulawska; Y = Yorkshire; P = Pietrain.

**Table 2:** ICP-MS method operating conditions and measurement parameters.

Parameters	Settings
RF power (W)	1500
Argon plasma gas flow (L/min)	15
Argon auxiliary gas flow rate (L/min)	0.8
Nebulizer gas flow rate (mL/min)	0.4
Spray chamber temperature (°C)	2.7
Helium flow rate in collision cell (mL/min)	5.0
Mass resolution	300
Dwell time for Se and As (s)	0.5
Dwell time for all elements, except Se and As (s)	0.1
Acquisition points for each mass	3
Replicates for peak integration	3

**Table 3:** ICP-MS multi-element method validation parameters.

Element	Measurement Mode ^1^	Internal Standard	LOD (mg/L)	LOQ (mg/L)	Repeatability (RSD)		R (%)
					Standard ^2^	Real Sample	
^7^Li	STD	^9^Be	0.001	0.003	1.3	5.5	106
^23^Na	KED	^45^Sc	0.876	2.65	3.5	0.8	78
^24^Mg	KED	^45^Sc	0.306	0.928	5.0	0.5	75
^31^P	KED	^45^Sc	2.41	7.29	1.0	1.6	113
^39^K	KED	^45^Sc	1.57	4.77	2.7	1.1	115
^44^Ca	KED	^45^Sc	5.22	15.8	0.2	1.3	90
^51^V	KED	^103^Rh	0.002	0.005	3.2	11.6	109
^52^Cr	KED	^103^Rh	0.003	0.009	1.1	2.1	112
^55^Mn	KED	^103^Rh	0.014	0.042	1.4	2.2	98
^57^Fe	KED	^103^Rh	0.290	0.879	6.0	2.3	107
^59^Co	KED	^103^Rh	0.001	0.003	1.7	5.9	117
^60^Ni	KED	^103^Rh	0.015	0.046	1.0	2.4	110
^63^Cu	KED	^103^Rh	0.009	0.027	1.8	1.4	97
^66^Zn	KED	^103^Rh	0.176	0.534	1.2	0.9	111
^71^Ga	KED	^103^Rh	0.001	0.002	1.1	10.4	95
^75^As	KED	^103^Rh	0.001	0.005	1.7	9.0	86
^78^Se	KED	^103^Rh	0.009	0.027	4.5	3.0	102
^85^Rb	KED	^103^Rh	0.003	0.010	1.8	1.3	112
^88^Sr	KED	^103^Rh	0.010	0.031	1.7	1.5	116
^95^Mo	KED	^103^Rh	0.007	0.020	1.1	1.2	83
^107^Ag	KED	^103^Rh	0.001	0.003	0.6	1.1	101
^111^Cd	KED	^103^Rh	0.0004	0.001	1.7	5.9	95
^133^Cs	KED	^103^Rh	0.0002	0.001	1.3	0.4	99
^138^Ba	KED	^103^Rh	0.008	0.025	1.1	4.2	96
^203^Tl	STD	^209^Bi	0.0004	0.001	1.5	8.9	115
^208^Pb	STD	^209^Bi	0.006	0.019	5.8	10.2	111
^238^U	STD	^209^Bi	0.0002	0.001	6.4	7.8	115

^1^ STD = standard (no gas); KED = kinetic energy discrimination (with He gas); ^2^ standard = 1 μg/L for all elements with the exception of Na, Mg, P, K, and Ca; standard = 1 mg/L for Na, Mg, P, K, and Ca. LOD = limit of detection, LOQ = limit of quantification; RSD = relative standard deviation; R% = recovery.

**Table 4:** Mean ± standard deviations of stable isotope ratios measured in defatted dry matter (DFDM) and mineral element concentrations (expressed on a wet weight basis) in *longissimus thoracis et lumborum* (LTL) pork samples from different European countries.

	Denmark	Poland	Italy	Spain
n.	80	118	210	204
Stable Isotope Ratios				
δ^18^O (‰)	13.9 ± 0.6	14.0 ± 0.3	13.3 ± 1.5	16.0 ± 0.9
δ^2^H (‰)	−111 ± 2	−103 ± 4	−101 ± 8	−89 ± 3
δ^13^C (‰)	−22.7 ± 0.3	−22.4 ± 0.4	−18.8 ± 2.1	−20.5 ± 1.2
δ^15^N (‰)	2.8 ± 0.2	2.9 ± 0.1	3.9 ± 0.4	3.7 ± 1.1
δ^34^S (‰)	3.1 ± 0.6	0.5 ± 0.8	1.0 ± 0.7	2.2 ± 0.9
Mineral Elements				
*Macronutrients*				
K (mg/kg)	3928 ± 140	3954 ± 642	3974 ± 356	3737 ± 469
P (mg/kg)	2004 ± 61	2086 ± 343	2093 ± 252	2002 ± 224
Na (mg/kg)	394 ± 27	380 ± 70	443 ± 60	373 ± 42
Mg (mg/kg)	237 ± 7.5	252 ± 42	267 ± 24	244 ± 24
Ca (mg/kg)	42.8 ± 6.39	42.9 ± 9.4	35.6 ± 4.97	38.2 ± 6.76
*Micronutrients*				
Zn (mg/kg)	9.60 ± 0.72	11.5 ± 2.29	14.2 ± 2.6	15.9 ± 5.1
Fe (mg/kg)	3.46 ± 0.39	4.20 ± 0.97	3.90 ± 0.73	5.72 ± 2.15
Cu (μg/kg)	414 ± 147	369 ± 75.8	356 ± 46.1	393 ± 85.4
Se (μg/kg)	110 ± 9.71	108 ± 22.9	144 ± 47.4	147 ± 42.3
Mn (μg/kg)	59 ± 6.85	66 ± 15	52.3 ± 11.4	62.8 ± 15.4
Cr (μg/kg)	21.6 ± 19.1	43.3 ± 58	11.6 ± 11.7	19.4 ± 33.6
Mo (μg/kg)	6.04 ± 3.59	7.17 ± 3.72	5.86 ± 3.01	7.04 ± 4.28
Ni (μg/kg)	3.75 ± 2.15	7.83 ± 6.49	4.16 ± 3.1	4.97 ± 2.89
Co (μg/kg)	0.35 ± 0.14	0.56 ± 0.26	0.62 ± 0.48	0.66 ± 0.3
*Non-Essential Elements*				
Rb (mg/kg)	4.52 ± 0.32	2.89 ± 0.67	5.29 ± 1.33	4.42 ± 0.95
Sr (μg/kg)	42.0 ± 11.8	23.5 ± 12.8	26.4 ± 13.7	33.6 ± 19.6
Ba (μg/kg)	29.7 ± 5.66	14.0 ± 4.74	22.4 ± 7.98	26.9 ± 15.6
Cs (μg/kg)	9.93 ± 18.6	34.1 ± 51.1	14 ± 9.28	22.6 ± 25.2
Pb (μg/kg)	1.11 ± 2.26	4.96 ± 6.73	1.89 ± 1.57	3.4 ± 4.73
Ag (μg/kg)	1.31 ± 0.93	1.12 ± 1.87	0.92 ± 0.9	0.55 ± 0.58
Li (μg/kg)	1.00 ± 0.42	1.67 ± 0.96	1.38 ± 1.47	1.71 ± 1.41
As (μg/kg)	0.64 ± 0.23	0.50 ± 0.2	0.51 ± 0.32	1.80 ± 1.42
Cd (μg/kg)	0.22 ± 0.05	0.39 ± 0.3	0.95 ± 1.2	1.00± 0.93
V (μg/kg)	0.36 ± 0.18	0.70 ± 0.58	0.4 ± 0.24	0.99 ± 0.79
Tl (ng/kg)	204 ± 42.1	737 ± 390	195 ± 168	288 ± 636
Ga (ng/kg)	41.6 ± 24.7	166 ± 438	113 ± 57.5	202 ± 246
U (ng/kg)	23 ± 17.8	39.4 ± 23.8	63.0 ± 72	76.6 ± 30.9

Italic text indicates the grouping of mineral elements into macronutrients, micronutrients, and non-essential elements.

**Table 5:** Mean values of stable isotope ratios measured in DFDM and mineral elements concentrations (expressed on a wet weight basis) in LTL pork samples from Poland.

	FARM				
	p1	p2	p3	Pooled SEM	Sign. *p*
n.	59	29	30		
Stable Isotope Ratios					
δ^18^O (‰)	13.7 ^b^	14.1 ^a^	14.2 ^a^	0.06	***
δ^2^H (‰)	−107 ^c^	−98 ^a^	−104 ^b^	0.37	***
δ^13^C (‰)	−22.5 ^b^	−21.9 ^a^	−22.7 ^b^	0.03	***
δ^15^N (‰)	2.9 ^b^	2.8 ^b^	3.1 ^a^	0.04	***
δ^34^S (‰)	1.2 ^a^	0.8 ^b^	−0.4 ^c^	0.12	***
Mineral Elements					
*Macronutrients*					
K (mg/kg)	3680 ^b^	4161 ^a^	4249 ^a^	9.98	**
P (mg/kg)	1928 ^b^	2219 ^a^	2241 ^a^	7.25	**
Na (mg/kg)	339 ^b^	428 ^a^	408 ^a^	3.10	**
Mg (mg/kg)	234 ^b^	270 ^a^	268 ^a^	2.52	**
Ca (mg/kg)	40.7 ^b^	44.7 ^a^	44.8 ^a^	1.04	**
*Micronutrients*					
Zn (mg/kg)	10.6 ^b^	10.9 ^b^	13.6 ^a^	0.30	**
Fe (mg/kg)	3.91 ^b^	4.32 ^a^	4.61 ^a^	0.14	**
Cu (μg/kg)	348 ^b^	362 ^a,b^	412 ^a^	3.05	**
Se (μg/kg)	115	100	102	1.65	n.s.
Mn (μg/kg)	60.3 ^b^	71.1 ^a^	71.5 ^a^	1.29	**
Cr (μg/kg)	11.5 ^c^	82.8 ^a^	62.6 ^b^	1.05	**
Mo (μg/kg)	6.82 ^b^	8.47 ^a^	6.56 ^b^	0.43	**
Ni (μg/kg)	3.79 ^c^	15.6 ^a^	8.13 ^b^	0.44	**
Co (μg/kg)	0.51 ^b^	0.67 ^a^	0.55 ^a,b^	0.12	**
*Non-Essential Elements*					
Rb (mg/kg)	2.62 ^b^	2.85 ^a,b^	3.42 ^a^	0.01	**
Sr (μg/kg)	24.5	23.5	21.8	0.77	n.s.
Ba (μg/kg)	24.1 ^b^	78.2 ^a^	8.78 ^c^	0.93	**
Cs (μg/kg)	14.0 ^b^	11.0 ^b^	16.7 ^a^	0.59	**
Pb (μg/kg)	3.53 ^b^	2.61 ^b^	9.80 ^a^	0.35	**
Ag (μg/kg)	0.96 ^b^	1.62 ^a^	0.97 ^b^	0.17	**
Li (μg/kg)	2.10 ^a^	0.97 ^c^	1.56 ^b^	0.20	**
As (μg/kg)	0.38 ^c^	0.50 ^b^	0.71 ^a^	0.11	**
Cd (μg/kg)	0.41 ^b^	0.19 ^c^	0.57 ^a^	0.10	**
V (μg/kg)	0.35 ^b^	0.91 ^a^	1.10 ^a^	0.13	**
Tl (ng/kg)	1049 ^a^	275 ^c^	631 ^b^	140	**
Ga (ng/kg)	148 ^b^	158 ^b^	206 ^a^	64.8	**
U (ng/kg)	31.5 ^b^	40.2 ^a,b^	52.8 ^a^	27.7	**

Italic text indicates the grouping of minerals into macronutrients, micronutrients, and non-essential elements. Different superscript letters within a row indicate significant differences among groups. Significance levels: ** *p* < 0.01; *** *p* < 0.001, n.s. not significant.

**Table 6:** Mean values of stable isotope ratios measured in DFDM and mineral elements concentrations (expressed on a wet weight basis) in LTL pork samples from Italy.

	FARM					
	I1	I2	i3	i4	Pooled	Sign. *p*
n.	20	30	59	100	SEM	
Stable Isotope Ratios						
δ^18^O (‰)	13.1 ^b^	15.3 ^a^	13.2 ^b^	11.6 ^c^	0.06	***
δ^2^H (‰)	−105 ^c^	−92 ^a^	−97 ^b^	−110 ^d^	0.25	***
δ^13^C (‰)	−18.0 ^b^	−18.5 ^b^	−17.0 ^a^	−21.9 ^c^	0.05	***
δ^15^N (‰)	3.6 ^b^	4.4 ^a^	3.7 ^b^	3.7 ^b^	0.02	***
δ^34^S (‰)	1.6 ^a^	1.4 ^a^	0.0 ^b^	1.0 ^a^	0.08	***
Mineral Elements						
*Macronutrients*						
K (mg/kg)	3826 ^b^	4080 ^a^	4064 ^a^	3939 ^a,b^	37.1	**
P (mg/kg)	1904 ^b^	2127 ^a^	2127 ^a^	2118 ^a^	26.5	**
Na (mg/kg)	451	450	449	438	6.01	n.s.
Mg (mg/kg)	264 ^b^	279 ^a^	273 ^a^	263 ^b^	2.44	**
Ca (mg/kg)	39.3 ^a^	37.3 ^a,b^	35.2 ^b^	34.5 ^b^	0.50	**
*Micronutrients*						
Zn (mg/kg)	14.7 ^a,b^	14.8 ^a,b^	15.1 ^a^	13.6 ^b^	0.28	**
Fe (mg/kg)	4.47 ^a,b^	4.66 ^a^	3.88 ^b^	3.56 ^c^	0.06	**
Cu (μg/kg)	375	373	354	347	4.95	n.s.
Se (μg/kg)	131 ^b^	104 ^c^	199 ^a^	124 ^b^	4.58	**
Mn (μg/kg)	48.0 ^b^	63.8 ^a^	49.1 ^b^	51.6 ^b^	1.13	**
Cr (μg/kg)	13.7	16.2	10.5	10.8	1.24	n.s.
Mo (μg/kg)	5.37 ^a,b^	6.34 ^a^	4.79 ^b^	6.36 ^a^	0.33	**
Ni (μg/kg)	4.83	5.22	3.93	3.70	0.33	n.s.
Co (μg/kg)	0.42 ^b^	1.54 ^a^	0.44 ^b^	0.49 ^b^	0.03	**
*Non-Essential Elements*						
Rb (mg/kg)	5.11 ^b^	4.09 ^c^	6.72 ^a^	4.76 ^b,c^	0.13	**
Sr (μg/kg)	27.8 ^a,b^	38.2 ^a^	23.0 ^b^	24.9 ^b^	1.40	**
Ba (μg/kg)	16.7	15.2	12.8	13.8	1.01	n.s.
Cs (μg/kg)	20.7 ^a,b^	21.0 ^b^	27.8 ^a^	19.4 ^b^	0.80	**
Pb (μg/kg)	2.71 ^a^	2.13 ^a^	2.18 ^a^	1.52 ^b^	0.17	**
Ag (μg/kg)	0.57	1.11	0.73	0.91	0.10	n.s.
Li (μg/kg)	1.13 ^b^	3.96 ^a^	0.88 ^b^	1.01 ^b^	0.11	**
As (μg/kg)	0.87 ^a^	1.08 ^a^	0.37 ^b^	0.37 ^b^	0.02	**
Cd (μg/kg)	1.23 ^a^	1.05 ^b^	0.48 ^c^	1.08 ^b^	0.13	**
V (μg/kg)	0.61 ^a^	0.51 ^a^	0.53 ^a^	0.33 ^b^	0.03	**
Tl (ng/kg)	126 ^b,c^	245 ^b^	354 ^a^	100 ^c^	2.01	**
Ga (ng/kg)	145	122	112	106	6.18	n.s.
U (ng/kg)	128 ^a^	74.3 ^a,b^	78.6 ^a,b^	40.2 ^b^	7.37	**

Italic text indicates the grouping of minerals into macronutrients, micronutrients, and non-essential elements. Different superscript letters within a row indicate significant differences among groups. Significance levels: ** *p* < 0.01; *** *p* < 0.001, n.s. not significant.

**Table 7:** Mean values of stable isotope ratios measured in DFDM and mineral elements concentrations (expressed on a wet weight basis) in LTL pork samples from Spain.

	FARM						
	S1	S2	s1	s3	s4	Pooled	Sign. *p*
n.	32	34	34	34	66	SEM	
Stable Isotope Ratios							
δ^18^O (‰)	15.9 ^c^	15.8 ^c^	17.2 ^a^	14.8 ^d^	16.6 ^b^	0.09	***
δ^2^H (‰)	−90 ^b,c^	−85 ^a^	−90 ^b^	−91 ^b,c^	−91 ^c^	0.32	***
δ^13^C (‰)	−20.7 ^b^	−21.4 ^b,c^	−19.4 ^a^	−21.9 ^c^	−19.2 ^a^	0.06	***
δ^15^N (‰)	4.0 ^b^	4.7 ^a^	3.1 ^c^	4.7 ^a^	2.1 ^d^	0.05	***
δ^34^S (‰)	2.8 ^a^	2.7 ^a^	2.6 ^a^	2.3 ^a^	0.7 ^b^	0.12	***
Mineral Elements							
*Macronutrients*							
K (mg/kg)	3444 ^d^	3054 ^e^	3661 ^c^	3877 ^b^	4181 ^a^	40.4	**
P (mg/kg)	1886 ^c^	1694 ^d^	1999 ^b^	2166 ^a^	2128 ^a^	25.5	**
Na (mg/kg)	384 ^a,b^	361 ^b^	405 ^a^	401 ^a^	343 ^c^	5.10	**
Mg (mg/kg)	232 ^b^	209 ^c^	246 ^a,b^	257 ^a^	258 ^a^	2.67	**
Ca (mg/kg)	39.6 ^a^	40.5 ^a^	39.1 ^a^	40.7 ^a^	34.7 ^b^	1.01	**
*Micronutrients*							
Zn (mg/kg)	16.8 ^b^	18.0 ^b^	15.7 ^b^	23.6 ^a^	10.5 ^c^	0.53	**
Fe (mg/kg)	6.89 ^b^	7.27 ^b^	4.66 ^c^	8.37 ^a^	3.58 ^d^	0.24	**
Cu (μg/kg)	436 ^a^	469 ^a^	381 ^b^	453 ^a,b^	312 ^c^	9.59	**
Se (μg/kg)	105 ^d^	118 ^c^	128 ^b^	129 ^b^	201 ^a^	1.98	**
Mn (μg/kg)	62.2 ^a,b^	65.4 ^a^	53.5 ^b^	67.3 ^a^	64.3 ^a^	2.48	**
Cr (μg/kg)	22.2 ^a,b^	38.6 ^a^	16.5 ^b^	18.5 ^b^	10.7 ^c^	5.22	**
Mo (μg/kg)	6.05	6.96	7.49	8.13	6.79	0.68	n.s.
Ni (μg/kg)	4.35	5.33	5.60	5.95	4.31	0.45	n.s.
Co (μg/kg)	0.67 ^a,b^	0.71 ^a^	0.74 ^a^	0.88 ^a^	0.47 ^b^	0.04	**
*Non-Essential Elements*							
Rb (mg/kg)	3.96 ^b^	3.02 ^c^	4.84 ^a,b^	3.98 ^b^	5.35 ^a^	0.08	**
Sr (μg/kg)	31.9	33.8	31.9	33.3	35.4	3.15	n.s.
Ba (μg/kg)	16.8 ^c^	29.5 ^a^	18.1 ^b,c^	29.1 ^a^	21.2 ^b^	3.93	**
Cs (μg/kg)	45.3 ^a^	16.3 ^b^	42.4 ^a^	17.8 ^b^	19.7 ^b^	1.95	**
Pb (μg/kg)	5.17 ^a^	4.13 ^a,b^	1.95 ^b^	5.17 ^a^	2.00 ^b^	0.74	**
Ag (μg/kg)	0.47 ^b^	0.89 ^a^	0.61 ^a,b^	0.34 ^b^	0.49 ^b^	0.09	**
Li (μg/kg)	2.75 ^a^	1.36 ^b^	1.18 ^c^	0.92 ^c^	2.02 ^a,b^	0.20	**
As (μg/kg)	0.89 ^d^	2.53 ^b^	0.59 ^e^	3.95 ^a^	1.43 ^c^	0.21	**
Cd (μg/kg)	0.81 ^b,c^	1.53 ^a^	1.00 ^a,b^	1.61 ^a^	0.53 ^c^	0.14	**
V (μg/kg)	1.07 ^a^	1.06 ^a^	1.02 ^a^	1.09 ^a^	0.85 ^b^	0.13	**
Tl (ng/kg)	260 ^b^	73.4 ^d^	284 ^b^	141 ^c^	482 ^a^	9.23	**
Ga (ng/kg)	229 ^a,b^	163 ^b,c^	142 ^c^	285 ^a^	196 ^b^	39.6	**
U (ng/kg)	77.0 ^a,b^	80.4 ^a,b^	97.9 ^a^	81.2 ^a,b^	61.5 ^b^	4.52	**

Italic text indicates the grouping of minerals into macronutrients, micronutrients, and non-essential elements. Different superscript letters within a row indicate significant differences among groups. Significance levels: ** *p* < 0.01; *** *p* < 0.001, n.s. not significant.

## Data Availability

The data presented in this study are openly available in FigShare at 10.6084/m9.figshare.31410264.

## Supplementary Materials

The following supporting information can be downloaded at: https://www.mdpi.com/article/10.3390/foods15081317/s1, Table S1. Mean values and standard deviations (SD) of stable isotope ratios (δ^18^O, δ^2^H, δ^13^C, δ^15^N, δ^34^S) and mineral element concentrations in LTL pork samples from Denmark, grouped by slaughter period. Table S2. Mean values and standard deviations (SD) of stable isotope ratios (δ^18^O, δ^2^H, δ^13^C, δ^15^N, δ^34^S) and mineral element concentrations in LTL pork samples from Poland, grouped by farm and breed. Table S3. Mean values and standard deviations (SD) of stable isotope ratios (δ^18^O, δ^2^H, δ^13^C, δ^15^N, δ^34^S) and mineral element concentrations in LTL pork samples from semi-intensive farms in Italy, grouped by farm and breed. Table S4. Mean values and standard deviations (SD) of stable isotope ratios (δ^18^O, δ^2^H, δ^13^C, δ^15^N, δ^34^S) and mineral element concentrations in LTL pork samples from intensive farms in Italy, grouped by farm and slaughter period. Table S5. Mean values and standard deviations (SD) of stable isotope ratios (δ^18^O, δ^2^H, δ^13^C, δ^15^N, δ^34^S) and mineral element concentrations in LTL pork samples from extensive farms in Spain, grouped by farm and breed. Table S6. Mean values and standard deviations (SD) of stable isotope ratios (δ^18^O, δ^2^H, δ^13^C, δ^15^N, δ^34^S) and mineral element concentrations in LTL pork samples from intensive farms in Spain, grouped by farm and breed. Table S7. Loadings, variance, and cumulative variance percentage for PCA of mineral elements data (Rotated Component Matrix). PCA loadings values higher than |0.4| are in bold. Table S8. Loadings, variance, and cumulative variance percentage for PCA of stable isotope ratio and mineral elements data (Rotated Component Matrix). PCA loadings values higher than |0.7| are in bold.

## Abbreviations

The following abbreviations are used in this manuscript:

PDO	Protected Designation of Origin
PGI	Protected Geographical Indication
SIRA	Stable Isotope Ratio Analysis
IRMS	Isotope Ratio Mass Spectroscopy
Q-ICP-MS	Single-Quadrupole Inductively Coupled Plasma Mass Spectrometer
PCA	Principal Component Analysis
ANOVA	Analysis of Variance
DU	Duroc
LA	Landrace
Y	Yorkshire
LW	Large White
PLW	Pulawska
P	Pietrain
MR	Mora Romagnola
CS	Cinta Senese
IB	Iberian
LTL	*Longissimus thoracis et lumborum*
DM	Dry matter
DFDM	Defatted Dry Matter
δ	Delta Notation
VPDB	Vienna Pee Dee Belemnite
VCDT	Vienna Canyon Diablo Troilite
VSMOW	Vienna Standard Mean Ocean Water
LOD	Limit of Detection
LOQ	Limit of Quantification
RSD	Relative Standard Deviation
R	Recovery
