# Supplementary material for: Multi-Country Study of Stable Isotopes and Mineral Elements in European Pork
Source: Foods. 2026 Apr 10;15(8):1317. doi: 10.3390/foods15081317 (PMC13114749; doi:10.3390/foods15081317)
Supplement: Supplementary file 1 [file foods-15-01317-s001.zip › foods-4197646-supplementary.pdf]

Supplementary materials

A Multi-country Study of Stable Isotopes and Mineral Elements in European Pork

Table S1. Mean values and standard deviations (SD) of stable isotope ratios ($\delta^{18}O$, $\delta^{2}H$, $\delta^{13}C$, $\delta^{15}N$, $\delta^{34}S$) and mineral element concentrations in LTL pork samples from Denmark grouped by slaughter period.

**Country**	**DENMARK**			
**FARM**	**d1**			
**System**	**Intensive indoor**			
**Main diet**	C_3			
**Breed**	**DU X (LAxY)**			
**Age at slaugher**	**6 months**			
**Slaughter period**	**May**		**Nov**	
**n**	**40**		**40**	
	**Mean**	**SD**	**Mean**	**SD**
Isotopic ratios				
**$\delta^{18}O$ (‰)**	14.16	0.58	13.64	0.55
**$\delta^{2}H$ (‰)**	-111	1.60	-111	1.54
**$\delta^{13}C$ (‰)**	-23.1	0.07	-22.4	0.10
**$\delta^{15}N$ (‰)**	2.91	0.16	2.69	0.14
**$\delta^{34}S$ (‰)**	2.90	0.59	3.35	0.45
Mineral elements				
Macronutrients				
K **(mg/Kg)**	3963	103	3894	159
P **(mg/Kg)**	2017	52.5	1991	65.1
Na **(mg/Kg)**	405	28.3	386	23.92
Mg **(mg/Kg)**	237	7.1	237	7.47
Ca **(mg/Kg)**	43.9	6.8	42.1	6.14
Micronutrients				
Zn **(mg/Kg)**	9.39	0.66	9.78	0.71
Fe **(mg/Kg)**	3.47	0.42	3.46	0.38
Cu **(µg/Kg)**	412	152	417	150
Se **(µg/Kg)**	117	6.97	102	5.92
Mn **(µg/Kg)**	59.5	6.25	58.6	7.84
Cr **(µg/Kg)**	14.03	14.14	29.81	21.01
Mo **(µg/Kg)**	6.35	3.52	5.42	3.07
Ni **(µg/Kg)**	3.68	2.39	3.83	1.94
Co **(µg/Kg)**	0.34	0.14	0.34	0.14
Non-essential elements				
Rb **(mg/Kg)**	4.38	0.24	4.66	0.35
Sr **(µg/Kg)**	41.95	12.18	41.57	10.76
Cs **(µg/Kg)**	25.18	2.40	34.65	3.61
Ba **(µg/Kg)**	11.34	19.75	9.04	18.70
Pb **(µg/Kg)**	1.24	2.86	1.06	1.70
Ag **(µg/Kg)**	0.79	0.34	1.78	1.03
Li **(µg/Kg)**	1.17	0.47	0.86	0.33

As **(µg/Kg)**	0.84	0.16	0.45	0.11
Cd **(µg/Kg)**	0.22	0.05	0.21	0.05
V **(µg/Kg)**	0.30	0.17	0.43	0.19
Tl **(ng/Kg)**	207	44.2	197	38.3
Ga **(ng/Kg)**	38.10	20.65	46.90	28.99
U **(ng/Kg)**	19.30	14.56	26.10	19.29

Table S2. Mean values and standard deviations (SD) of stable isotope ratios ($\delta^{18}O$, $\delta^{2}H$, $\delta^{13}C$, $\delta^{15}N$, $\delta^{34}S$) and mineral element concentrations in LTL pork samples from Poland, grouped by farm and breed.

**Country**	**POLAND**											
**FARM**	**p1**								**p2**		**p3**	
**System**	**intensive indoor**											
**Main diet**	C_3											
**Breed**	**LW (POL)**		**LW(POL) X PLW**		**PLW**		**PIC**		**(LWxLA) X (DUxP)**		**PLW**	
**Age at slaughter**	**5.5**		**5.7**		**6**		**5**		**5**		**7**	
**Slaughter period**	**Jan**						**Feb**		**May**		**Oct**	
**n**	**10**		**9**		**10**		**30**		**29**		**30**	
	**Mean**	**SD**	**Mean**	**SD**	**Mean**	**SD**	**Mean**	**SD**	**Mean**	**SD**	**Mean**	**SD**
Isotopic ratios												
**$\delta^{18}O$ (‰)**	13.6	0.1	13.8	0.3	14.0	0.3	13.5	0.3	14.1	0.3	14.2	0.4
**$\delta^{2}H$ (‰)**	-107	1.3	-110	1.6	-110	1.2	-105	1.7	-99	1.2	-103	2.3
**$\delta^{13}C$ (‰)**	-22.7	0.1	-22.8	0.2	-22.8	0.0	-22.3	0.1	-21.9	0.1	-22.7	0.1
**$\delta^{15}N$ (‰)**	2.64	0.17	2.50	0.15	2.68	0.27	3.19	0.17	2.82	0.14	3.06	0.15
**$\delta^{34}S$ (‰)**	0.52	0.39	0.47	0.54	0.79	0.65	1.73	0.67	0.79	0.68	-0.42	0.74
Mineral elements												
Macronutrients												
K **(mg/Kg)**	3493	287	3714	409	4016	506	3675	982	4151	382	4259	338
P **(mg/Kg)**	1748	207	1866	226	2023	263	1996	496	2207	215	2246	177
Na **(mg/Kg)**	320	51	332	55	348	41	342	92	424	46	405	34
Mg **(mg/Kg)**	220	22	231	28	248	30	236	65	268	25	268	20
Ca **(mg/Kg)**	44	9.4	38	7.0	43	5.6	38	9.4	44	8.6	45	8.9
Micronutrients												
Zn **(mg/Kg)**	11.0	0.2	11.4	1.4	11.8	0.4	10.0	2.6	10.9	1.3	13.7	1.9
Fe **(mg/Kg)**	3.9	0.3	4.2	0.5	5.3	0.4	3.7	1.2	4.3	0.7	4.6	0.7
Cu **(µg/Kg)**	323	49	344	42	423	42	342	97	359	31	415	81
Se **(µg/Kg)**	134	7.2	144	13.3	145	4.6	95	26.8	100	6.7	103	12.0
Mn **(µg/Kg)**	55.0	5.1	62.2	10.3	70.5	11.8	58.7	17.3	69.3	8.9	70.9	16.0
Cr **(µg/Kg)**	6.51	2.39	3.63	5.44	8.53	6.17	15.91	18.57	90.43	88.69	62.83	50.85
Mo **(µg/Kg)**	5.91	4.68	6.33	3.42	7.94	2.43	6.44	2.91	8.64	2.30	6.69	3.57
Ni **(µg/Kg)**	4.04	0.84	2.89	2.63	4.42	2.31	3.58	2.35	16.06	5.94	8.13	4.83
Co **(µg/Kg)**	0.559	0.160	0.466	0.286	0.594	0.162	0.442	0.216	0.624	0.181	0.528	0.236
Non-essential elements												
Rb **(mg/Kg)**	2.64	0.20	3.02	0.30	3.20	0.17	2.41	0.67	2.85	0.26	3.41	0.74
Sr **(µg/Kg)**	22.7	20.2	26.1	5.2	22.2	4.0	21.0	14.1	20.8	8.3	21.2	8.6
Cs **(µg/Kg)**	17.7	3.6	19.4	4.6	19.1	1.0	10.2	4.1	10.9	1.1	16.7	3.1
Ba **(µg/Kg)**	64.6	23.7	32.2	70.3	34.2	47.7	6.5	4.7	84.2	75.6	7.8	3.8
Pb **(µg/Kg)**	1.72	0.63	1.36	0.72	1.87	0.77	5.07	5.90	2.70	0.85	10.19	10.33
Ag **(µg/Kg)**	0.37	0.10	0.10	0.44	0.24	0.13	1.04	1.33	1.68	2.34	0.94	0.90
Li **(µg/Kg)**	1.92	1.33	2.36	0.58	1.14	0.30	2.17	0.89	0.93	0.49	1.43	0.35
As **(µg/Kg)**	0.35	0.05	0.31	0.12	0.34	0.08	0.42	0.16	0.50	0.09	0.69	0.20
Cd **(µg/Kg)**	0.37	0.61	0.40	0.26	0.43	0.42	0.39	0.24	0.18	0.06	0.58	0.28
V **(µg/Kg)**	0.35	0.09	0.23	0.16	0.35	0.16	0.39	0.25	0.96	0.49	1.03	0.64
Tl **(ng/Kg)**	1050	238	988	145	772	175	1127	299	274	43	621	200
Ga **(ng/Kg)**	76.4	6.8	34.3	54.5	55.6	27.7	73.6	37.5	165.1	113.1	171.2	182.9
U **(ng/Kg)**	31.7	13.7	29.9	15.3	21.8	10.0	35.3	17.5	37.7	20.2	52.3	33.0

Table S3. Mean values and standard deviations (SD) of stable isotope ratios ($\delta^{18}O$, $\delta^{2}H$, $\delta^{13}C$, $\delta^{15}N$, $\delta^{34}S$) and mineral element concentrations in LTL pork samples from semi extensive farms in Italy, grouped by farm and breed.

**Country**	**ITALY**									
**FARM**	**I1**				**I2**					
**System**	**Semi - extensive**				**Semi - extensive**					
**Main diet**	**C_3 and C_4**				**C_3 and C_4**					
**Breed**	**LW X D**		**MR**		**CS**		**LW X CS**		**D X CS**	
**Age at slaugher**	**10**		**14**		**13**		**13-14**		**13-14**	
**Slaughter period**	**Jun**		**Jul**		**Jul**		**Jul-Aug**		**Jul-Aug**	
**n**	10		10		10		10		10	
	**Mean**	**SD**	**Mean**	**SD**	**Mean**	**SD**	**Mean**	**SD**	**Mean**	**SD**
Isotopic ratios										
**$\delta^{18}O$ (‰)**	13.1	0.3	13.2	0.4	15.5	0.3	15.3	0.4	15.0	0.4
**$\delta^{2}H$ (‰)**	-106	0.7	-103	0.5	-92	1.3	-90	1.9	-94	2.0
**$\delta^{13}C$ (‰)**	-18.2	0.1	-17.8	0.1	-18.4	0.1	-18.6	0.4	-18.5	0.3
**$\delta^{15}N$ (‰)**	3.18	0.20	3.95	0.19	4.35	0.20	4.41	0.22	4.54	0.13
**$\delta^{34}S$ (‰)**	1.83	0.82	1.42	0.85	1.39	0.76	1.33	0.78	1.49	0.39
Mineral elements										
Macronutrients										
K **(mg/Kg)**	3940	217	3726	158	4119	258	3965	426	4195	428
P **(mg/Kg)**	1783	120	2027	74	2115	196	2135	453	2211	402
Na **(mg/Kg)**	439	47	460	47	408	29	451	38	494	42
Mg **(mg/Kg)**	260	21	269	20	272	16	279	21	287	21
Ca **(mg/Kg)**	42.7	5.5	36.3	4.2	33.6	3.8	37.6	4.6	40.2	3.9
Micronutrients										
Zn **(mg/Kg)**	10.9	0.8	18.3	1.5	14.0	1.4	15.1	2.0	15.9	2.4
Fe **(mg/Kg)**	3.88	0.46	5.04	0.62	4.69	0.34	4.71	0.54	4.64	0.27
Cu **(µg/Kg)**	333	25.4	419	46.9	365	18.2	356	31.5	402	64.2
Se **(µg/Kg)**	111	4.6	151	13.1	115	5.7	100	15.6	101	13.6
Mn **(µg/Kg)**	53.7	9.8	43.7	9.4	65.7	11.8	63.5	6.2	62.2	7.4
Cr **(µg/Kg)**	21.6	12.5	5.9	7.2	21.8	17.4	14.5	15.8	11.7	9.0
Mo **(µg/Kg)**	5.02	2.05	5.80	0.60	6.75	1.15	7.01	3.01	4.15	1.89
Ni **(µg/Kg)**	6.54	2.76	3.27	2.44	5.26	4.04	5.16	3.79	5.93	3.71
Co **(µg/Kg)**	0.397	0.135	0.450	0.057	2.161	0.802	1.199	0.249	1.360	0.195
Non-essential elements										
Rb **(mg/Kg)**	5.72	0.32	4.54	0.29	4.14	1.26	4.05	1.00	4.27	0.95
Sr **(µg/Kg)**	28.2	11.3	28.4	14.9	35.8	16.4	33.8	7.0	44.3	13.0
Cs **(µg/Kg)**	24.8	3.4	17.0	4.1	19.5	13.4	21.5	10.3	23.2	12.4
Ba **(µg/Kg)**	23.5	22.2	10.3	5.7	9.6	3.2	18.9	5.5	17.9	5.7
Pb **(µg/Kg)**	2.71	1.46	2.76	1.94	2.73	2.10	1.86	1.80	1.64	1.31
Ag **(µg/Kg)**	0.739	0.328	0.355	0.183	0.589	0.132	1.683	1.653	1.105	1.017
Li **(µg/Kg)**	1.76	0.48	0.51	0.21	2.37	1.69	4.15	2.19	5.27	1.29
As **(µg/Kg)**	0.773	0.192	0.968	0.255	1.052	0.319	1.082	0.233	1.213	0.295
Cd **(µg/Kg)**	1.71	1.49	0.70	1.01	1.11	1.35	0.86	0.82	1.33	1.35
V **(µg/Kg)**	0.86	0.29	0.37	0.13	0.62	0.22	0.34	0.16	0.57	0.28
Tl **(ng/Kg)**	96.6	50.0	144.2	27.9	193.4	61.9	232.8	98.5	310.8	100.1
Ga **(ng/Kg)**	179	17	113	40	100	0	145	136	113	36
U **(ng/Kg)**	187	10	69	91	55	34	56	40	82	99

Table S4. Mean values and standard deviations (SD) of stable isotope ratios (δ^{18}O, δ^{2}H, δ^{13}C, δ^{15}N, δ^{34}S) and mineral element concentrations in LTL pork samples from intensive farms in Italy, grouped by farm and slaughter period.

**Country**	**ITALY**											
**FARM**	**i3**				**i4**							
**System**	**intensive indoor**				**intensive with oudoor access**							
**Main diet**	**C_3 and C_4**				**organic C_3**							
**Breed**	**LW X D**				**TOPIGS**							
**Age at slaugher**	**10**				**10**							
**Slaughter period**	**Jul.**		**Jan.**		**Aug.**		**Feb.**		**Oct.**		**May**	
**n**	**30**		**30**		**20**		**30**		**20**		**30**	
	**Mean**	**SD**	**Mean**	**SD**	**Mean**	**SD**	**Mean**	**SD**	**Mean**	**SD**	**Mean**	**SD**
Isotopic ratios												
**δ^{18}O (‰)**	13.4	0.7	12.9	0.3	11.7	0.4	11.9	0.4	11.0	0.5	11.5	0.5
**δ^{2}H (‰)**	-96	1.6	-99	1.0	-107	1.1	-109	1.6	-114	1.8	-110	1.6
**δ^{13}C (‰)**	-17.2	0.1	-16.7	0.1	-21.3	0.2	-22.2	0.1	-22.6	0.1	-21.4	0.1
**δ^{15}N (‰)**	3.79	0.17	3.65	0.16	3.73	0.14	3.69	0.20	3.75	0.21	3.70	0.18
**δ^{34}S (‰)**	0.73	0.82	-0.66	0.57	0.96	0.80	1.09	0.49	1.25	0.47	0.93	0.55
Mineral elements												
Macronutrients												
K **(mg/Kg)**	4025	281	4123	270	4049	260	3735	532	4290	245	3811	216
P **(mg/Kg)**	2056	254	2200	141	2189	139	2030	308	2310	140	2041	134
Na **(mg/Kg)**	474	85	428	46	473	40	399	68	482	39	419	28
Mg **(mg/Kg)**	283	32	265	15	275	13	255	26	275	16	246	14
Ca **(mg/Kg)**	37.1	4.1	33.0	3.9	35.5	4.3	32.0	4.5	37.3	4.4	34.7	2.6
Micronutrients												
Zn **(mg/Kg)**	15.0	2.6	15.1	2.6	13.1	1.3	13.0	1.7	15.0	2.9	13.1	1.7
Fe **(mg/Kg)**	4.24	0.61	3.52	0.47	3.46	0.48	3.38	0.59	3.90	0.47	3.57	0.70
Cu **(μg/Kg)**	350	29.2	363	39.9	319	41.8	335	40.2	386	39.3	358	45.1
Se **(μg/Kg)**	152	36.3	243	20.1	108	8.2	118	10.7	136	8.2	138	10.4
Mn **(μg/Kg)**	47.0	10.5	51.8	9.4	47.0	7.4	52.3	9.7	61.3	11.6	48.4	8.1
Cr **(μg/Kg)**	11.4	10.5	9.6	7.1	8.8	7.0	6.7	8.9	18.0	17.7	8.1	6.5
Mo **(μg/Kg)**	3.74	1.13	5.23	2.65	5.87	1.71	4.69	2.20	8.37	4.28	6.93	2.99
Ni **(μg/Kg)**	4.88	2.46	3.18	3.06	5.16	3.20	2.85	1.57	2.89	1.33	4.39	3.59
Co **(μg/Kg)**	0.511	0.163	0.389	0.161	0.440	0.114	0.533	0.217	0.404	0.134	0.542	0.176
Non-essential elements												
Rb **(mg/Kg)**	5.66	0.42	7.79	0.59	4.48	0.34	4.31	0.59	4.45	0.35	5.85	0.47
Sr **(μg/Kg)**	26.1	15.9	21.0	15.5	21.8	11.3	24.7	9.1	26.4	11.5	26.0	13.0
Cs **(μg/Kg)**	20.9	2.0	34.0	4.1	18.7	2.9	17.3	2.2	14.7	1.9	26.8	3.4
Ba **(μg/Kg)**	13.6	9.0	11.4	8.0	13.2	8.9	13.1	8.3	14.3	6.7	13.8	7.6
Pb **(μg/Kg)**	2.63	1.89	1.66	1.46	1.23	0.84	1.01	0.80	2.20	1.53	1.82	1.47
Ag **(μg/Kg)**	0.767	0.341	0.690	0.355	0.957	1.173	0.809	0.599	0.598	0.544	1.563	1.415
Li **(μg/Kg)**	0.74	0.41	0.91	0.47	1.95	0.94	0.57	0.42	0.92	0.73	0.78	0.62
As **(μg/Kg)**	0.409	0.114	0.334	0.072	0.373	0.120	0.432	0.139	0.330	0.076	0.317	0.119
Cd **(μg/Kg)**	0.20	0.09	0.69	0.90	1.49	1.65	1.19	1.00	0.25	0.28	1.65	1.59
V **(μg/Kg)**	0.51	0.23	0.55	0.19	0.26	0.12	0.27	0.15	0.41	0.26	0.36	0.13
Tl **(ng/Kg)**	196	51.0	488	10	18.4	24.6	19.8	27.2	285	67.3	114	42.8
Ga **(ng/Kg)**	122	52	104	22	100	0	106	30	107	31	108	40
U **(ng/Kg)**	121	84	46	48	39	22	33	36	60	68	28	29

Table S5. Mean values and standard deviations (SD) of stable isotope ratios ($\delta^{18}O$, $\delta^{2}H$, $\delta^{13}C$, $\delta^{15}N$, $\delta^{34}S$) and mineral element concentrations in LTL pork samples from extensive farms in Spain, grouped by farm and breed.

**Country**	SPAIN							
**FARM**	S1				S2			
**System**	Extensive (Montanera)				Extensive (Cebo de Campo)			
**Main diet**	Grass, acorns (C_3)				Grass, acorns, feed (C_3)			
**Breed**	IB		IBXD		IB		IBXD	
**Age at slaugher**	14		14		23		23	
**Slaughter period**	Jan		Feb		Mar		Mar	
n	17		17		17		17	
	**Mean**	**SD**	**Mean**	**SD**	**Mean**	**SD**	**Mean**	**SD**
Isotopic ratios								
**$\delta^{18}O$ (‰)**	15.9	0.3	16.0	0.4	16.1	0.2	15.5	0.6
**$\delta^{2}H$ (‰)**	-90	2.2	-91	2.1	-84	0.9	-86	1.4
**$\delta^{13}C$ (‰)**	-20.1	0.2	-21.4	0.1	-21.5	0.2	-21.4	0.1
**$\delta^{15}N$ (‰)**	4.12	0.23	3.95	0.24	4.96	0.30	4.57	0.30
**$\delta^{34}S$ (‰)**	3.08	0.56	2.44	0.63	2.69	0.83	2.86	0.94
Mineral elements								
Macronutrients								
K **(mg/Kg)**	3352	276	3553	216	2972	263	3123	222
P **(mg/Kg)**	1844	162	1960	145	1641	139	1747	140
Na **(mg/Kg)**	386	26	388	26	366	25	356	25
Mg **(mg/Kg)**	229	23	237	13	202	17	213	14
Ca **(mg/Kg)**	38.8	5.6	40.4	6.2	41.0	10.3	40.7	6.6
Micronutrients								
Zn **(mg/Kg)**	18.6	2.9	15.1	2.0	18.9	2.7	17.5	3.5
Fe **(mg/Kg)**	8.20	1.20	5.39	0.64	8.39	0.98	6.54	0.93
Cu **(µg/Kg)**	444	60.6	438	73.9	514	85.9	442	53.8
Se **(µg/Kg)**	105	5.9	107	7.4	125	12.3	111	9.6
Mn **(µg/Kg)**	67.4	18.6	54.6	12.9	73.5	11.3	60.4	10.3
Cr **(µg/Kg)**	27.9	30.5	12.6	14.6	80.7	86.4	10.2	4.0
Mo **(µg/Kg)**	6.94	3.67	4.75	3.94	7.59	5.28	6.64	2.90
Ni **(µg/Kg)**	4.22	2.05	4.33	3.10	7.05	3.23	4.16	1.59
Co **(µg/Kg)**	0.778	0.311	0.513	0.246	0.776	0.351	0.692	0.279
Non-essential elements								
Rb **(mg/Kg)**	3.90	0.55	4.00	0.29	2.89	0.22	3.15	0.32
Sr **(µg/Kg)**	35.4	21.2	30.2	16.4	30.4	21.9	28.2	13.5
Cs **(µg/Kg)**	53.3	21.5	37.4	19.7	13.7	6.3	18.9	8.9
Ba **(µg/Kg)**	17.5	8.2	16.3	8.6	31.5	20.3	23.9	9.1
Pb **(µg/Kg)**	4.93	5.88	3.99	6.32	4.45	4.37	3.50	3.36
Ag **(µg/Kg)**	0.577	0.654	0.352	0.605	1.049	1.153	0.845	0.622
Li **(µg/Kg)**	4.58	2.31	0.89	0.71	1.43	0.59	1.25	0.34
As **(µg/Kg)**	1.125	0.368	0.666	0.221	2.240	0.749	2.674	1.737
Cd **(µg/Kg)**	0.98	0.99	0.63	0.30	1.04	0.51	1.87	0.94
V **(µg/Kg)**	1.48	1.88	0.66	0.25	1.37	0.39	0.82	0.28
Tl **(ng/Kg)**	328.3	78.6	180.7	36.6	70.1	16.2	72.3	22.1
Ga **(ng/Kg)**	345	387	113	61	191	77	133	55
U **(ng/Kg)**	90	30	64	18	88	34	65	16

Table S6. Mean values and standard deviations (SD) of stable isotope ratios ($\delta^{18}O$, $\delta^{2}H$, $\delta^{13}C$, $\delta^{15}N$, $\delta^{34}S$) and mineral element concentrations in LTL pork samples from intensive farms in Spain, grouped by farm and breed.

**Country**	**SPAIN**					
**FARM**	**s1**		**s3**		**s4**	
**System**	**intensive**		**intensive**		**intensive**	
**Main diet**	**C_3 and C_4**		**C_3**		**C_3 and C_4**	
**Breed**	**IBXD**		**IB**		**L X LW**	
**Age at slaugher**	**12**		**50**		**7**	
**Slaughter period**	**May**		**Jan**		**Feb**	
**n**	**34**		**34**		**68**	
	**Mean**	**SD**	**Mean**	**SD**	**Mean**	**SD**
Isotopic ratios						
**$\delta^{18}O$ (‰)**	17.1	0.5	14.8	0.6	16.6	0.6
**$\delta^{2}H$ (‰)**	-90	1.1	-91	1.6	-91	2.6
**$\delta^{13}C$ (‰)**	-19.4	0.2	-21.9	0.1	-19.2	0.3
**$\delta^{15}N$ (‰)**	3.11	0.20	4.64	0.23	2.06	0.26
**$\delta^{34}S$ (‰)**	2.54	0.78	2.39	0.57	0.60	0.71
Mineral elements						
Macronutrients						
K **(mg/Kg)**	3645	222	3869	313	4187	214
P **(mg/Kg)**	1988	118	2159	192	2130	143
Na **(mg/Kg)**	398	34	398	35	340	34
Mg **(mg/Kg)**	245	15	258	22	258	13
Ca **(mg/Kg)**	38.1	6.2	40.0	6.5	34.6	4.1
Micronutrients						
Zn **(mg/Kg)**	15.4	2.0	23.7	3.1	10.5	1.3
Fe **(mg/Kg)**	4.53	0.54	8.35	1.18	3.54	0.42
Cu **(µg/Kg)**	373	41.6	449	56.4	311	47.0
Se **(µg/Kg)**	126	9.6	129	11.3	201	22.3
Mn **(µg/Kg)**	51.8	12.0	66.9	20.7	64.2	13.0
Cr **(µg/Kg)**	8.0	4.0	14.4	15.0	10.7	16.4
Mo **(µg/Kg)**	6.64	3.73	7.50	3.99	6.82	3.78
Ni **(µg/Kg)**	5.38	2.12	5.79	3.91	4.23	2.69
Co **(µg/Kg)**	0.660	0.199	0.849	0.215	0.469	0.189
Non-essential elements						
Rb **(mg/Kg)**	4.78	0.38	3.96	0.51	5.34	0.46
Sr **(µg/Kg)**	29.2	13.2	34.8	25.8	35.7	15.7
Cs **(µg/Kg)**	41.0	6.2	17.7	4.1	19.6	3.2
Ba **(µg/Kg)**	16.5	7.7	27.9	18.1	21.7	39.0
Pb **(µg/Kg)**	1.93	1.60	3.87	3.72	1.98	2.36
Ag **(µg/Kg)**	0.599	0.342	0.318	0.183	0.478	0.594
Li **(µg/Kg)**	1.11	0.50	0.87	0.51	1.98	1.06
As **(µg/Kg)**	0.587	0.153	3.797	1.069	1.420	0.658
Cd **(µg/Kg)**	1.03	0.64	1.67	1.56	0.53	0.39
V **(µg/Kg)**	0.93	0.26	1.08	0.77	0.85	0.67
Tl **(ng/Kg)**	277.6	78.1	140.3	42.0	389.2	904.2
Ga **(ng/Kg)**	129	121	306	339	181	253
U **(ng/Kg)**	95	33	81	25	60	25

Table S7. Loadings, variance, and cumulative variance percentage for PCA of mineral elements data (Rotated Component Matrix). PCA loadings values higher than |0.4| are in bold.

Variable	Component	
	PC1	PC2
Mg	**0.915**	0.002
Na	**0.810**	-0.094
K	**0.829**	0.159
P	**0.799**	0.168
Fe	**0.623**	0.014
Zn	**0.786**	0.268
Se	0.399	**0.842**
Rb	0.395	**0.846**
Sr	**-0.423**	-0.175
Cs	-0.376	**0.753**
Tl	0.061	**0.842**
Variance %	40.6	26.0
Cumulative %	40.6	66.6

Table S8. Loadings, variance, and cumulative variance percentage for PCA of stable isotope ratio and mineral elements data (Rotated Component Matrix). PCA loadings values higher than |0.7| are in bold.

Variable	Component			
	PC1	PC2	PC3	PC4
Mg	**0.871**	0.334	-0.059	0.010
Na	**0.837**	0.129	0.225	-0.104
K	**0.844**	0.322	-0.314	0.062
P	**0.842**	0.280	-0.160	0.056
V	-0.220	0.098	0.140	**0.815**
Mn	0.212	-0.162	-0.120	**0.749**
Fe	-0.271	-0.100	**0.798**	0.294
Zn	0.116	0.212	**0.886**	0.067
Ga	-0.115	0.048	0.129	**0.820**
Se	0.211	**0.846**	-0.169	0.032
Rb	0.445	**0.761**	-0.054	-0.098
$\delta^{15}N$	0.013	-0.135	**0.913**	-0.105
$\delta^{13}C$	0.044	**0.891**	0.194	0.023
$\delta^{18}O$	**-0.717**	0.282	-0.093	0.293
Variance %	29.9	16.2	15.7	13.9
Cumulative %	29.9	46.1	61.9	75.7
